# Financing of Human Papillomavirus Vaccination in Africa: A Scoping Review

**DOI:** 10.1002/hsr2.73254

**Published:** 2026-09-25

**Authors:** Kafayat Aminu, Jimoh Amzat, Rita Amarachi Nwebo, Precious Chika Nnannah, Chiamaka Norah Ezeagu, Aboyowa Arayuwa Edukugho, Olubukola Omobowale, Abba Shehu, Yovanthi Anurangi Jayasinghe, Ganiyat Amzat, Kehinde Kazeem Kanmodi

**Affiliations:** ^1^ Centre for Evidence Synthesis and Implementation Research Cephas Health Research Initiative Inc Ibadan Nigeria; ^2^ Department of Sociology Usmanu Danfodiyo University Sokoto Nigeria; ^3^ Department of Sociology University of Johannesburg Johannesburg South Africa; ^4^ Centre for Digital Health Research Innovation and Practice, Cephas Health Research Initiative Inc Ibadan Nigeria; ^5^ The African Field Epidemiology Network Nigeria Country Office Abuja Nigeria; ^6^ Department of Community Medicine University of Ibadan Ibadan Nigeria; ^7^ Department of Research and Innovation University of Technology and Entrepreneurship Phnom Penh Cambodia; ^8^ Department of Economics Usmanu Danfodiyo University Sokoto Nigeria; ^9^ Office of the Executive Director Cephas Health Research Initiative Inc Ibadan Nigeria; ^10^ Department of Public Health Thomas Adewumi University Oko Nigeria; ^11^ Department of Community Dentistry University of the Witwatersrand Johannesburg South Africa

**Keywords:** Africa, financing, human papillomavirus, review, vaccination

## Abstract

**Background and Aims:**

Human papillomavirus (HPV) vaccination is a critical intervention for cervical cancer prevention in Africa; however, program uptake and scale‐up remain limited due to financing constraints. This scoping review mapped existing evidence on HPV vaccine financing in Africa, identifying prevailing funding mechanisms, cost estimates, delivery strategies, and challenges and opportunities for sustainable financing.

**Methods:**

Guided by the Arksey and O'Malley framework and PRISMA‐ScR guidelines, we systematically searched nine electronic databases for peer‐reviewed studies. Retrieved records were deduplicated and screened against eligibility criteria. Eligible studies underwent data extraction, followed by collation, summarization, and thematic synthesis. All costs were standardized to United States (US) Dollars and further adjusted to a common reference year (2025US) using the US Consumer Price Index (CPI‐U) to enable comparison across study years.

**Results:**

Twenty‐seven peer‐reviewed studies were included. The review found heavy dependence on donor support, particularly from Gavi, with limited domestic co‐financing across most countries. Per‐dose costs ranged from US$0.27–US$19.76 (financial) and US$1.31–US$45.00 (economic) as reported, or US$0.32–US$26.50 and US$1.63–US$60.36 after adjustment to 2025 US; per fully immunized girl, costs ranged from US$6.68–US$40.03 and US$17.31–US$91.19 as reported, or US$8.31–US$53.69 and US$21.53–US$122.31 adjusted. Primary cost drivers included service delivery, personnel, and outreach to out‐of‐school girls. School‐based delivery was more cost‐effective in several contexts, and the single‐dose schedule consistently showed lower cost with comparable efficacy, enhancing cost‐effectiveness and reducing programmatic burden. Willingness to pay exists among private individuals, but out‐of‐pocket models remain inequitable.

**Conclusion:**

Donor funds, mainly Gavi, still drive HPV vaccination in most of Africa. The single‐dose schedule cuts costs and logistics while remaining effective. For equitable, sustainable access, African countries must prioritize cost‐effective vaccines, boost domestic funding, strengthen health systems, and adopt models that lessen reliance on external support.

## 1 Introduction

Cervical cancer remains a leading cause of cancer‐related mortality among women globally, with low‐ and middle‐income countries (LMICs), particularly in sub‐Saharan Africa, bearing the highest burden. Human papillomavirus (HPV), a sexually transmitted infection, is the primary etiological agent for nearly all cases of cervical cancer [1, 2, 3]. The introduction of HPV vaccines represents a major breakthrough in global public health, offering a preventive tool to significantly reduce the incidence and mortality associated with cervical cancer [4, 5]. Despite this, the uptake and sustained implementation of HPV vaccination programs across Africa have been limited, with financing emerging as a major barrier to equitable access and long‐term program sustainability [6].

Africa accounts for approximately 19 of the 20 countries with the highest cervical cancer burdens globally [7, 8]. Although the WHO launched the global strategy to eliminate cervical cancer as a public health problem—aiming to vaccinate 90% of girls by age 15, screen 70% of women, and treat 90% of those diagnosed—many African countries lag significantly behind these targets [3, 9]. HPV vaccine financing challenges intersect with broader systemic issues in health financing constraints across the continent [10], including heavy reliance on donor funding, weak fiscal capacity, competing health priorities, and limited domestic budget allocations for immunization [11]. The cost of HPV vaccines, infrastructure demands, human resource needs, and communication campaigns collectively place substantial financial pressure on national immunization programs. Although Gavi, the Vaccine Alliance, has played a critical role in subsidizing vaccine procurement and supporting demonstration projects in low‐income countries, its support is time‐bound and based on eligibility thresholds [11, 12].

Domestic financing mechanisms for HPV vaccination vary widely across African countries and are often constrained by limited health budgets, dependence on out‐of‐pocket payments, and weak public health insurance systems. Countries like Rwanda and South Africa have demonstrated successful national rollouts through strong political will, strategic donor alignment, and integration into school‐based health platforms [13, 14]. However, these remain exceptions rather than the norm. For most African nations, scaling HPV vaccination beyond small pilot programs requires not only political commitment but also durable financing models that can absorb costs over time, especially in the face of competing demands such as malaria, HIV, and maternal and child health services. In addition to procurement costs, long‐term financing must address health systems strengthening, including cold chain capacity, community mobilization, surveillance, and workforce training. The failure to integrate HPV vaccination into broader health system financing strategies often leads to vertical programming approaches, which may be unsustainable and inefficient [15]. Furthermore, the COVID‐19 pandemic has disrupted immunization services and reallocated fiscal resources, further constraining progress on HPV vaccine delivery and financing [16].

Existing reviews have effectively catalogued the *costs* of HPV vaccination programs [17, 18]. However, understanding the cost of a program differs from understanding how such programs are financed. This review fills a distinct gap by adding *financing*—investigating the flow of funds, and the roles of different actors (e.g., governments, donors). Hence, this scoping review aims to map existing literature on the financing of HPV vaccination programs in Africa, identify key funding sources, challenges, and strategies, and highlight best practices and gaps in the evidence base. By synthesizing information across peer‐reviewed articles, grey literature, and programmatic reports, the review seeks to inform policy development and guide future research on sustainable HPV vaccine financing models in Africa. Understanding the financial landscape is critical for enabling African countries to make informed decisions, develop resilient funding mechanisms, and ultimately reduce the burden of cervical cancer among their populations.

## 2 Methods

### 2.1 Study Design

This study utilized the scoping review method established by Arksey and O'Malley—a widely recognized method for conducting scoping reviews [19]. To follow the best practice guidelines for scoping reviews, this review was conducted and reported following the Preferred Reporting Items for Systematic Reviews and Meta‐Analyses extension for Scoping Reviews (PRISMA‐ScR) [20].

### 2.2 Identification of the Research Question

The research question guiding this review was: “What are the current financing mechanisms for HPV vaccination in Africa, and what challenges and opportunities exist for improving funding and access?” This research question was designed to provide a comprehensive understanding of various funding models, financial strategies, and broader financial landscape for HPV vaccination in Africa, while also identifying challenges and opportunities for enhancing access and sustainability.

### 2.3 Identification of Relevant Literature

**Figure 1:**
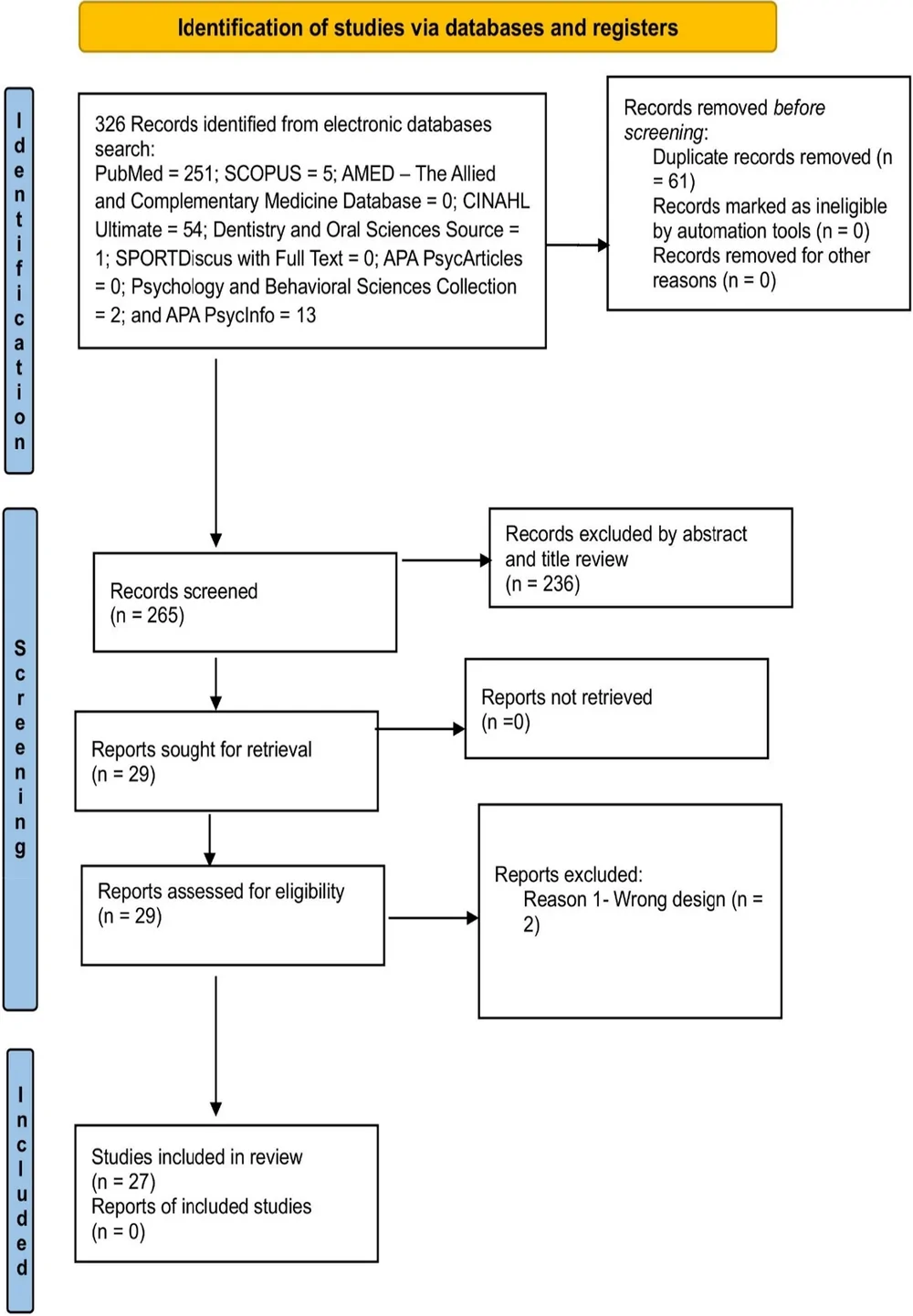
PRISMA flow diagram highlighting the included studies in compliance with PRISMA 2020 guidelines.

A systematic search of nine electronic research databases was conducted to gather all literature records relevant to financing mechanisms for HPV vaccination in Africa was done on the 9th of December 2024. The databases searched included SCOPUS, PubMed, CINAHL Ultimate, APA PsyArticles, SPORTDiscus with Full Text, The Allied and Complementary Medicine Database (AMED), Dentistry and Oral Sciences Source, Psychology and Behavioral Sciences Collection, and APA PsycInfo (Figure 1). The search incorporated relevant terms—which were obtained from Thesaurus and Medical subject Heading (MeSH) Dictionary—such as “money”, “grant*”, “fund*”, “sponsor*”, “financ*”, “loan”, “bank*”, “budget*”, “aid”, “donor”, “donat*”, “invest*”, “allocat*”, “Human papilloma*”,“HPV”, “vaccin*”, “immun*”, “Algeria”, “Angola”, “Benin”, “Botswana”, “Burkina Faso”, “Burundi” “Cameroon”, “Central African Republic”, “Chad”, “Ivory coast”, “Cote d'Ivoire”, “Djibouti”, “Democratic Republic of Congo”, “Egypt”, “Equitorial guinea”, “Eritrea”, “Eswatini”, “Ethiopia”, “Gabon”, “Gambia”, “Ghana”, “Guinea”, “Guinea‐Bissau”, “Kenya”, “Lesotho”, “Liberia”, “Libya”, “Madagascar”, “Malawi “Mauritania”, “Mauritius”, “Mali”, “Mauritania”, “Morocco”, “Mozambique”, “Namibia”, “Niger”, “Nigeria”, “Rwanda”, “Sao Tome and Principe”, “Senegal”, “Seychelles”, “Sierra Leone”, “Somalia”, “South Africa”, “Sudan”, “Tanzania”, “Togo, “Tunisia”, “Uganda”, “Zambia”, “Zimbabwe”, “Reunion”, “Saint Helena”, “Western Sahara”, “Mayotte.” Boolean operators (“OR” and “AND”) were combined in the search to obtain relevant literature records. Tables S1–S3 of the supplementary file contain the search strings used for the search of each of the selected databases.

### 2.4 Selection of Relevant Literature

The literature selection process was done using Rayyan software [21]. Rayyan is a web‐based tool which is used for deduplicating and screening literature in systematic reviews [21]. This software allows systematic reviewers to work collaboratively on it during deduplicating, literature screening, conflict resolution, and decision tracking [21]. The benefits of using this software for systematic reviews are that it makes the review process faster, organized, and collaborative [21]. However, its limitation is that it is not ideal for large or complex systematic reviews.

To streamline the selection process of this review, the duplicates from the compiled literature records were identified and eliminated using Rayyan (Figure 1). Following deduplication, the remaining literature records underwent comprehensive screening to evaluate their suitability for inclusion based on a set of predetermined eligibility criteria (Figure 1). The selection process of literature adhered to the following inclusion criteria to ensure that only the relevant empirical research studies on the financing of HPV vaccination in Africa were included in this review:

- Peer‐reviewed journal articles (i.e., literature that underwent thorough review by experts in the field).
- Empirical studies that specifically examined issues related to the financing of HPV vaccination in Africa. Examples of these issues include budgeting, out‐of‐pocket financing, fundraising, funding allocation, funding sources, financial challenges, and cost‐effectiveness.
- Studies on individuals, population groups or entities in Africa. These entities include banks, loan sharks, cooperative societies, multilateral organizations, bilateral organizations, national organizations, local organizations, and private organizations.
- Journal articles published in English.

The screening process was two‐staged. The first stage involved the examination (at face value) of the titles and abstracts of all deduplicated literature records to identify and consider those records that met these inclusion criteria for full‐text screening (second stage); also, those records that it was unclear if they met the inclusion criteria of this review were considered for full‐text screening to avoid inadvertent exclusion of relevant articles from the review (Figure 1).

The second stage of the screening process involved the full‐text retrieval and detailed examination of all literature records retained in the first, to confirm their suitability for inclusion into the review (Figure 1). At this stage, any literature that did not meet all the inclusion criteria of the review was excluded (Table S4 [Supplementary File]). Notably, three independent reviewers (R.A.N., P.C.N., and K.A.) were involved in the screening process, and at least two reviewers (R.A.N. and P.C.N.) screened each literature record in each stage. In a situation where conflicts exist in the decisions of two reviewers, regarding the inclusion or exclusion of a record/literature, they were resolved through consultation with a third reviewer (K.A.). Overall, this selection process was adopted to ensure that only relevant peer‐reviewed original research articles were included in this scoping review.

### 2.5 Quality Appraisal of Included Studies

We selected the Mixed Methods Appraisal Tool (MMAT), version 2018 by Hong et al. [22] to assess the methodological quality of the included studies. This tool was appropriate for evaluating the overall body of evidence comprising heterogeneous study designs and facilitated the assessment of the methodological quality of each individual study against standardized criteria [22]. The MMAT consists of two generic screening questions applicable to all study types, followed by five specific methodological criteria tailored to each study design category—qualitative, quantitative (randomized, non‐randomized, or descriptive), or mixed methods.

Each included article was independently assessed by two reviewers (YAJ and KKK) using a checklist specific to the study design. The reviewers rated each criterion as “Yes” (score = 1), “No” (score = 0), or “Can't tell” (score = 0.5), based on a three‐point scale, with a cumulative score ranging from 0 to 7. Any discrepancies in assessment were resolved through discussion between YAJ and KKK. A score of < 3.5 was rated as “Below average”, 3.5 as “Average” and > 3.5 as “Above average”. A summary of the risk of bias assessment is presented in Tables S5–S8 (Supplementary File).

### 2.6 Data Charting

Data charting in this scoping review involved systematically extracting relevant information from the included studies to address the research question. A structured form was devised to ensure consistency and comprehensiveness in the data charting process. The form was used to extract key elements such as study details (title, authors, year of publication, country), research objectives, methodology (study design, sample size, data collection methods), financing mechanisms (types of funding, sources, costs), outcomes (impact and cost‐effectiveness), and discussion on implications (policy, practice, future research) from the included articles. Notably, cost estimates were extracted exactly as reported in the original studies, including the stated currency and reference year. Because the included studies used different currencies and cost years, and sufficient economic parameters were not specified (such as price base year, inflation rate, or exchange rate), we did not convert the costs to a common reference currency or year.

Cost estimates were extracted and reported exactly as presented in the original studies, including the stated currency and reference year. Due to variations in costing frameworks, reporting formats, and the absence of sufficient economic parameters in several studies (e.g., base year, inflation indices, and exchange rates), the reported costs were not directly converted to a common currency year or inflation‐adjusted value. However, to facilitate temporal interpretation across studies conducted between 2008 and 2025, a supplementary analysis of the relative purchasing power of the United States Dollar (US$) across study years was presented separately using 2008 as the reference year. This approach minimized the introduction of potentially inaccurate assumptions while supporting contextual comparison of cost estimates across studies conducted in different periods.

Overall, the form was designed to capture detailed insights into the financing of HPV vaccination in Africa, allowing for a thorough analysis of funding models, challenges and opportunities. This process was carried out using Microsoft Word because of its simplicity and user‐friendly interface. Discrepancies were addressed by select team members (RAN, PCN, and KKK) to ensure consistency and accuracy in data charting.

### 2.7 Collation, Summary, and Reporting of Results

A thematic synthesis approach was employed in the collation, summarization, and reporting of the data charted in this review. This method facilitated the organization of extracted information into coherent themes [23, 24], offering a comprehensive understanding of the financing of HPV vaccination in Africa. The data were reviewed multiple times, coded, and grouped into broader categories reflecting significant themes (e.g., funding sources, economic and financial cost of HPV vaccine, disaggregation of economic and financial costs, investment and recurrent costs of HPV vaccine, willingness to pay for HPV vaccine, cost and mode of HPV vaccine delivery, impact and cost‐effectiveness). These themes were refined for accuracy and distinctiveness and validated by multiple team members. The final themes provided detailed insights into the financing mechanisms and their impact on HPV vaccination coverage.

### 2.8 Inflation‐Adjusted HPV Vaccination Cost Estimates (Base Year: 2025 US$)

To enable comparison of costs reported across studies conducted in different years, all extracted cost estimates (already denominated in US$ in the original studies) were additionally adjusted to a common reference year (2025 US$) using the U.S. Bureau of Labor Statistics Consumer Price Index for All Urban Consumers (CPI‐U), calendar‐year averages [25]. The reference (cost) year for each estimate was taken from the year explicitly stated in the source study where available; where a study did not report a specific cost year, the year was estimated as the publication year minus two, reflecting the typical lag between data collection and publication for costing studies, and this assumption is flagged in Table S10 (Supporting File S2). The formular for the adjustment is as follows:

Adjusted cost(base year)=Original cost×[CPI(base year)/CPI(original cost year)]

Both the originally reported values and the inflation‐adjusted values are presented, in order to assess relative cost differences across countries and delivery strategies independent of the year in which each study was conducted, while the original reported values are preserved for fidelity to the source studies. This adjustment does not correct for cross‐country differences in local inflation, exchange‐rate movements, or purchasing‐power parity, since original cost estimates were reported directly in US$ without the underlying local‐currency and exchange‐rate parameters needed for such adjustment; it therefore corrects only for the erosion of US$ purchasing power over time (2008–2025) [26, 27, 28].

## 3 Results

### 3.1 Focus of Studies on HPV Financing

As the summary in Table 1A–AE shows, 27 relevant studies were included in this review. Although their focus varied, some common thematic areas were identified. Nearly one‐quarter of the studies (*n* = 6/27, 22.2%) focused on planning and cost estimation/analysis of HPV vaccine programs [29, 30, 31, 32, 33, 34] (see Table 1A). Similarly, 22.2% (6/27) assessed the cost‐effectiveness, economic and health implications of HPV vaccination, including the long‐term value/sustainability of different vaccination strategies [35, 36, 37, 38, 39, 40] (see Table 1B). The operational cost challenges and delivery strategies for HPV vaccination were examined by 18.5% (5/27) [17, 41, 42, 43, 44] (see Table 1C). About 11.1% (3/27) explored public perception and willingness to pay for HPV vaccine [45, 46, 47] (Tables 1D), and 25.9% (7/27) focused on health system implications of HPV vaccination [48, 49, 50, 51, 52, 53] (Table 1E).

**Table 1A:** Summary of studies on planning and cost estimation/analysis of HPV vaccine programs in africa.

S/n	Author (s) and year	Research objective/s	Study design	Country	Type of data (primary or secondary)	Participants and sample size	Source of funding for the HPV vaccine	Estimated cost of HPV vaccine per dose (US$) [original in black; 2025 US$‐adjusted in blue]	Results related to the review objective/s and question/s	Conclusion/recommendation
1.	Alonso et al., (2019) [29]	To estimate the costs associated with the demonstration program of HPV vaccination during the 2014 cycle in the Manhiça district, and developed an alternative cost scenario for future implementation.	Mixed‐method Study	Mozambique	Primary	10‐year‐old girls enrolled in schools.	GAVI	$6.07 per dose **2025 US$ (inflation‐adjusted): FC/dose: $8.25; EC/dose: $23.92; FC/FIG: $24.41; EC/FIG: $71.11** *Reference year: 2014, Stated*	Cost per FIG: EC US$52.29, FC US$17.95; Cost per dose: EC US$17.59, FC US$6.07. Total project cost (3‐dose schedule): US$349,400 (vaccine price = US$5/dose).	A reduction in personnel costs (through restriction of the outreach vaccinator team or number of supervision visits) is necessary
2.	Brennan et al., (2022) [30]	To determine the cost of national HPV vaccination introduction in Senegal's routine immunization program for a single‐age cohort of nine‐year‐old girls	Cohort study (Retrospective)	Senegal	Primary	9‐year‐old girls.	GAVI, Senegal's ministry of health	Financial cost: $3.07 Economic cost: $7.56 **2025 US$ (inflation‐adjusted): FC/dose: $3.82; EC/dose: $9.40** *Reference year: 2020, Assumed*	The total financial and economic delivery costs of Senegal's HPV vaccination program was $1,152,351 and $2,838,466, respectively. Equivalent to ($ 3.07 and $ 7.56 per dose, respectively).	Findings would be useful for other countries planning to introduce the HPV vaccine and other new vaccines at a national scale
3.	Hidle et al., (2018) [31]	To determine the cost of Zimbabwe's human papillomavirus (HPV) vaccination demonstration project.	Quantitative Descriptive Study	Zimbabwe	Primary and secondary	10‐year‐old girls, 5724 fully immunized girls	GAVI, United States Centers for Disease Control and Prevention from contract number 200‐2015‐63464‐0001.	FC: $19.76 per dose EC: $45.00 per dose FC: $40.03 per FIG EC: $91.19 per FIG **2025 US$ (inflation‐adjusted): FC/dose: $26.50; EC/dose: $60.36; FC/FIG: $53.69; EC/FIG: $122.31** *Reference year: 2016, Assumed*	The FC per FIG: $5.34 (in schools), $34.90 (health facilities), and $288.63 (outreach). The EC per FIG: $17.39, $41.25, and $635.84, respectively.	Vaccinating overlapping groups of girls had the potential of reducing the financial costs per dose of HPV vaccine.
4.	Hutubessy, et al., (2012) [32]	To present the purpose, definitions, methods, data sources and assumptions behind the generic WHO C4P tool to aid planning and costing of nationwide HPV vaccination program in LMIC	Quantitative Descriptive Study	Tanzania	Primary and secondary	1.6 million fully vaccinated girls	Donations from the manufacturer, GAVI and Bill and Melinda Gates Foundation	Financial cost: $10.77 Economic cost: $12.24 **2025 US$ (inflation‐adjusted): FC/dose: $15.42; EC/dose: $17.52** *Reference year: 2011, Stated*	Program‐level costs: Establishing a school‐based HPV vaccination program (3 doses) was estimated at US$9.2 million (**excluding vaccine costs**) and US$31.5 million (including vaccine costs, assuming US$5 per dose).	Governments need to plan for non‐vaccine costs to ensure adequate funds for vaccine introduction.
5.	Ngabo et al., (2015) [33]	To understand the introduction and delivery cost per dose or child of the three new vaccines in Rwanda to inform domestic and external financial resource mobilization.	Quantitative Descriptive Study	Rwanda	Primary and secondary	12‐year‐old girls in Primary 6.	GAVI	Financial cost: $10.23 Economic cost: $14.45 **2025 US$ (inflation‐adjusted): FC/dose: $14.13; EC/dose: $19.96** *Reference year: 2013, Assumed*	The financial unit cost per fully immunized child of delivering 3 doses of each vaccine (**excluding vaccine costs**) was $10.23. Gardasil® had a higher total cost due to resource requirements for a new vaccine delivery system.	Service delivery strategies have an important influence on the costs of introducing new vaccines.
6.	Simuyemba et al., 2023 [34]	To establish the financial and economic costs associated with administering the HPV vaccine, particularly focusing on the costs of delivering a single dose	Quantitative Descriptive Study	Zambia	Secondary data supplemented by primary data	Girls aged 14 years (in‐school and out‐of‐school)	Gavi, the Vaccine Alliance.	Financial Cost: USD 0.55 per dose Overall FC: US$6.0 per dose; per FIG: US$11.9 EC: US$23.0 per dose and US$46.0 per FIG **2025 US$ (inflation‐adjusted): FC/dose: $7.13; EC/dose: $27.32; FC/FIG: $14.14; EC/FIG: $54.64** *Reference year: 2021, Assumed*	Vaccine delivery: School‐based HPV vaccine delivery in Zambia had the lowest economic cost at USD 13.2 per dose and USD 26.4 per fully immunized child,	Though Gavi currently supports HPV vaccination in Zambia, its long‐term sustainability is threatened by vaccine costs, necessitating the prioritisation of key cost drivers and the development of cost‐minimisation strategies.

Abbreviations: EC, economic cost; FC, financial cost; FIG, fully immunized girl.

**Table 1B:** Summary of studies on cost‐effectiveness, economic and health implications of HPV vaccination in Africa.

S/n	Author (s) and year	Research objective/s	Study design	Country	Type of data (primary or secondary)	Participants and sample size	Source of funding for the HPV vaccine	Estimated cost of HPV vaccine per dose (US$) [original in black; 2025 US$‐adjusted in blue]	Results related to the review objective/s and question/s	Conclusion/recommendation
1.	Campos et al., (2012) [31]	To determine the health and economic impact of HPV 16/18 vaccination and cervical cancer screening in Eastern Africa	Quantitative Descriptive Study	Kenya, Mozambique, Tanzania, Uganda, and Zimbabwe.	Secondary data	9–12 years old	GAVI, Bill and Melinda Gates Foundation and National Cancer Institute.	$2 to $54.25 per dose **2025 US$ (inflation‐adjusted): $2.95 – $80.07 per dose** *Reference year: 2010, Assumed*	Cost‐effectiveness: The study reveals that HPV 16/18 vaccination at 70% coverage could reduce the lifetime risk of cervical cancer by 40%. Vaccination cost under $10 is believed to be “very cost‐effective”, while between $10 and $25 is “good value”	Vaccination and screening could significantly reduce cervical cancer mortality in Eastern Africa. In resource‐poor settings, global financial support is central for implementing CC prevention and control.
2.	Goldie et al., (2008) [32]	To estimate the health and economic consequences expected with HPV 16,18 vaccination of young adolescent girls.	Quantitative Descriptive Study	72 GAVI‐eligible countries	Secondary data	72 GAVI‐eligible countries	GAVI	$2 per dose **2025 US$ (inflation‐adjusted): $3.19 per dose** *Reference year: 2006, Assumed*	Cost‐effectiveness: The cost per dosage has a significant impact on how cost‐effective the HPV vaccine is. However, the cost‐effectiveness of vaccines decreases dramatically as their prices rise.	Introducing the HPV vaccine to young adolescent girls in GAVI‐eligible countries would be highly cost‐effective if the vaccine is priced at US$ 2 per dose.
3.	Khiari et al., (2024) [33]	To assess the cost‐utility of the implementation of HPV vaccination program in Tunisia	Quantitative Descriptive Study	Tunisia	Secondary data	Women between 10 to 84 years.	GAVI	Cost per dose: US$45 **2025 US$ (inflation‐adjusted): Up to $49.49 per dose** *Reference year: 2022, Assumed*	Incremental cost‐effectiveness ratio (ICER): Adding vaccination to screening remained below the GDP per capita threshold; ICER US$2,910 − 3,245/QALY at vaccine prices up to US$45 per dose and 100% coverage.	Implementation of HPV vaccination in addition to ongoing control program would be cost‐effective
4.	Kiatpongsan and Kim, (2014) [34]	To determine a range of vaccine costs for which the 9‐valent vaccine would be cost‐effective in comparison to the current HPV vaccines.	Quantitative Descriptive Study	Kenya and Uganda	Secondary data	Pre‐adolescent girls	GAVI	Kenya: $9.7 per vaccinated girl, Uganda: $8.3 per vaccinated girl **2025 US$ (inflation‐adjusted): Kenya: $13.60 per vaccinated girl; Uganda: $11.64 per vaccinated girl** *Reference year: 2012, Assumed*	Compared to the 2‐ and 4‐valent vaccines, the 9‐valent vaccine is more expensive but provides more health benefits. Additional costs for the 9‐valent immunization were 71% and 61% more, in Kenya and Uganda than the current vaccine pricing.	Despite evidence of cost‐effectiveness, critical challenges around affordability and feasibility of HPV vaccination and other competing needs in low‐resource settings such as Kenya and Uganda remain.
5.	Kiendrébéogo et al., (2023) [35]	To estimate the potential health and economic impact of GARDASIL‐4® in Burkina Faso and consider alternative policy options to increase the value and/or impact of the current national HPV vaccination program.	Quantitative Descriptive Study	Burkina Faso	Secondary	10 cohorts of 9‐year‐old girls	GAVI	Not indicated *2025 US$: not applicable (no cost estimate reported)*	Cost‐effectiveness outcomes: CECOLIN® remained the most cost‐effective vaccine, with annual program costs of US$2.9 M (CECOLIN®), US$4.1 M (GARDASIL‐4®), US$4.4 M (CERVARIX®), and US$19.8 M (GARDASIL‐9®).	To improve the cost‐effectiveness of HPV vaccine, a single‐dose strategy and/or alternative Gavi‐supported HPV vaccines should be considered
6.	Messoudi et al., 2019 [36]	To assess the cost‐effectiveness of implementing an HPV vaccination program in Morocco, alongside the current screening method	Quantitative Descriptive Study	Morocco	Both primary and secondary data	Women (30‐49 years); Pre‐adolescent girls aged 14 years	Pan American Health Organization (PAHO) Revolving Fund	Initial HPV vaccine cost: $10 per dose, administration cost per dose: $5 Total cost: $15 per vaccinated girl (assuming a two‐dose schedule) **2025 US$ (inflation‐adjusted): Vaccine: $13.13/dose; Admin: $6.57/dose; Total: $19.70 per vaccinated girl (2‐dose)** *Reference year: 2017, Assumed*	Cost‐effectiveness: HPV vaccination for pre‐adolescent girls in Morocco is a highly cost‐effective strategy for reducing cervical cancer Vaccination alone is more cost‐effective than expanding screening coverage beyond 15%	Prioritizing HPV vaccination for pre‐adolescent girls in Morocco is the most cost‐effective cervical cancer prevention strategy.

**Table 1C:** Summary of studies on operational cost challenges and delivery strategies for HPV vaccination in Africa.

S/n	Author (s) and year	Research objective/s	Study design	Country	Type of data (primary or secondary)	Participants and sample size	Source of funding for the HPV vaccine	Estimated cost of HPV vaccine per dose (US$) [original in black; 2025 US$‐adjusted in blue]	Results related to the review objective/s and question/s	Conclusion/recommendation
1.	Slavkovsky et al., (2024) [17]	To estimate the potential cost savings for delivery and procurement costs under a single‐dose schedule.	Quantitative Descriptive Study	Ethiopia, Guyana, Rwanda, Sri Lanka, and Uganda	Primary and secondary data	30 to 66 health facilities, 5 to 29 subnational administrative offices, and 1 national office	Not indicated	Financial cost: $4.84 ‐ $12.34 per single dose and $9.64 ‐ $23.43 per 2 dose. Economic cost: $7.86 ‐ $28.53 per single dose **2025 US$ (inflation‐adjusted): FC: $5.32–$13.57 per single dose; $10.60–$25.77 per 2‐dose. EC: $8.64–$31.38 per single dose** *Reference year: 2022, Assumed*	One‐ vs two‐dose scenarios: The cost per dose is estimated to increase under a single‐dose schedule, whereas Cost per adolescent receiving the full schedule would decrease.	A single‐dose HPV vaccination schedule is likely to reduce Ongoing program to improve the likelihood of program affordability and sustainability.
2.	Burger et al., (2018) [37]	To evaluate the long‐term health and economic impacts of routine one‐dose HPV vaccination compared to [1] no vaccination and [2] two‐dose HPV vaccination in a low‐income country	Quantitative Descriptive Study	Uganda	Secondary data	9‐year‐old girls	GAVI	$6.90 per dose and $13.80 for 2 doses **2025 US$ (inflation‐adjusted): $9.25 per dose and $18.51 for 2 doses** *Reference year: 2016, Assumed*	Cost‐effectiveness: One‐dose HPV vaccination (70% coverage) prevented 15‐16% of cervical cancer cases while a 21% decline was recorded with two‐dose vaccination.	It is important to take factors such as feasibility, acceptability, Affordability, and value for money into account when selecting an HPV vaccination program.
3.	Mvundura et al., (2023) [38]	To understand the contextual and operational factors of HPV vaccine delivery in six national immunization programs that are past the introduction years and to estimate their ongoing delivery costs, from the perspective of the health system.	Mixed Methods Study	Ethiopia Guyana, Rwanda, Senegal, Sri Lanka, and Uganda	Primary and secondary		Gavi	Financial cost: $0.27 to $3.32 Economic cost: $3.09 (Rwanda) to $17.20 **2025 US$ (inflation‐adjusted): FC: $0.32 to $3.94. EC: $3.67 (Rwanda) to $20.43** *Reference year: 2021, Assumed*	Vaccine costs: Mean annual economic costs ranged from $1,207 to $3,190. At the national level, it ranged from $7,657 to $304,278.	There is a need for regionally specific cost data to inform a more nuanced understanding of the costs of HPV vaccine delivery.
4.	Quentin et al., (2012) [39]	To analyze the observed costs of school‐based vaccination project and explore cost differences between delivering vaccines to rural and urban schools and between the two alternative delivery strategies.	Quantitative Descriptive Study	Tanzania	Primary	4211 girls were vaccinated	GAVI	$5 per dose **2025 US$ (inflation‐adjusted): $7.38 per dose** *Reference year: 2010, Assumed*	Total economic project costs (3 doses) of HPV vaccine was US$349,400 (including a vaccine price of US$5 per dose). Class‐based vaccine delivery achieved lower cost per FIG compared to age‐based delivery.	Vaccine can be delivered at costs that would make HPV vaccination a very cost‐effective intervention.
5.	Vodicka et al., (2022) [40]	To evaluate the projected cost‐effectiveness and budget impact of adding HPV vaccine into Ghana's national immunization program	Quantitative Descriptive Study	Ghana	Secondary	9‐year‐old girls	GAVI, Ghana ministry of health, PATH, Bill and Melinda Gates foundation.	$4.50 ‐ $4.60 **2025 US$ (inflation‐adjusted): $5.60 – $5.72** *Reference year: 2020, Assumed*	Cost‐effective: National HPV vaccination in Ghana was projected to be cost‐effective compared to no vaccination in all scenarios evaluated.	Introducing HPV vaccination in Ghana would be cost‐effective compared to no vaccination, with the bivalent vaccine being the most cost‐effective strategy.

**Table 1D:** Summary of studies on public perception and willingness to pay for HPV vaccine in Africa.

s/n	Author (s) and year	Research objective/s	Study design	Country	Type of data (primary or secondary)	Participants and sample size	Source of funding for the HPV vaccine	Estimated cost of HPV vaccine per dose (US$) [original in black; 2025 US$‐adjusted in blue]	Results related to the review objective/s and question/s	Conclusion/recommendation
1.	Chigozie et al., (2022) [41]	We explored the willingness of men in Nigeria to encourage their partner/family members to take and pay for HPV vaccine and cervical cancer screening.	Cross‐sectional analytical study	Nigeria	Primary	352 participants	Private out‐of‐pocket payment	Not applicable *2025 US$: not applicable (no cost estimate reported)*	Willingness to pay: 59% of Nigerian men are willing to support HPV vaccination and cervical screening for their family members. However, the cost of HPV vaccine is a potential barrier to willingness to pay.	To improve HPV vaccine and screening access, increased health education and subsidized or co‐payment systems are crucial
2.	Marfo et al., (2024) [42]	To explore a current privately funded HPV vaccination program to identify practice challenges and gaps, and to gather insights to inform practice and national policy.	Qualitative descriptive study	Ghana	Primary	HPV vaccinators [8], Program/policy leaders [8]	Private out‐of‐pocket payment	Not applicable *2025 US$: not applicable (no cost estimate reported)*	Policy implications: Ghana requires stronger government financing and clearer policies for sustainable HPV vaccination	There is a need for a collective effort in HPV vaccination advocacy to stimulate political and government interests in policy and decision‐making
3.	Umeh, Nduka and Ekwunife, (2016) [43]	To assess Nigerian mothers’ willingness to pay for the HPV vaccine using a contingent valuation method.	Cross‐sectional analytical study	Nigeria	Primary	401 participants	Private out‐of‐pocket payment	$5.84 per dose **2025 US$ (inflation‐adjusted): $7.94 per dose** *Reference year: 2014, Assumed*	Willingness to pay: The average WTP was US$ 11.68. This is opposed to the estimated delivery cost of US$ 18.16 and US$ 19.26 for urban and rural populations respectively at the vaccine price offered by the Vaccine Alliance (Gavi) and US$ 35.16 and US$ 36.26 for urban and rural populations respectively	High demand for vaccines should be exploited to increase vaccine uptake. Co‐payment could be a feasible financing strategy in the event of national HPV vaccination programs.

**Table 1E:** Summary of studies on health system cost implications of HPV vaccination in Africa.

s/n	Author (s) and year	Research objective/s	Study design	Country	Type of data (primary or secondary)	Participants and sample size	Source of funding for the HPV vaccine	Estimated cost of HPV vaccine per dose (US$) [original in black; 2025 US$‐adjusted in blue]	Results related to the review objective/s and question/s	Conclusion/recommendation
1.	Biellik et al., (2009) [44]	To synthesize data from the health system structure component of the formative research to describe each individual health system and immunization financing structure.	Qualitative descriptive study	India, Peru, Uganda, and Vietnam	Primary	53 focus group discussions, 374 in‐depth or key informant interviews; consultations with immunization experts; 45 exit interviews; 10 facility assessments; 11 observations of vaccination sessions	PATH, Bill & Melinda Gates Foundation	$7 to $21 per immunized child depending on the vaccine components. **2025 US$ (inflation‐adjusted): $10.87 to $32.61 per immunized child** *Reference year: 2007, Assumed*	HPV vaccine introduction: Uganda's system demonstrates strong readiness for HPV vaccine introduction, though sustainability will require investment in logistics, training, and long‐term financing.	Low‐resource settings have the potential to successfully adopt the HPV vaccines, if the health system structures and immunization financing options are well understood.
2.	Portnoy et al., (2021) [49]	To evaluate the potential health, equity and FRP benefits of sustaining routine HPV vaccination in Ethiopia.	Quantitative Descriptive Study	Ethiopia	Secondary	14‐year‐old girls	GAVI, the World Bank and the World Health Organization (WHO)	$10.70 **2025 US$ (inflation‐adjusted): $13.47** *Reference year: 2019, Assumed*	Economic impact: The vaccination program of a single cohort of 14‐year‐old girls at 95% coverage exceeded $16 million but would avert $1.9 million ($1.4– 2.0 million) associated with CC treatment costs (1% of total costs compared to no vaccination)	Routine HPV vaccination needs to be prioritized once the global vaccine shortage is overcome. The results can inform fair and efficient priority settings in low‐ and middle‐income countries with similar demographic and economic characteristics.
3.	Levin et al., (2022) [46]	To prevent and control cervical cancer which includes projected costs of scaling up secondary and tertiary interventions of cervical cancer in addition to vaccination as part of the global elimination strategy	Quantitative Descriptive Study	Tanzania	Primary	14‐year‐old girls	WHO	Financial cost: $6.68 Economic cost: $17.31 **2025 US$ (inflation‐adjusted): FC/FIG: $8.31; EC/FIG: $21.53** *Reference year: 2020, Stated*	Projected national program costs (2020–2024): Financial cost (FC) US$68 million; Economic cost (EC) US$124 million. Per‐FIG FC US$6.68, EC US$17.31.	Scaling up cervical cancer prevention and control will reduce morbidity and mortality over time
4.	Hidle et al., (2022) [45]	To document the incremental resource needs for national HPV vaccination introduction at district and health facility administrative levels and estimate the cost of resources used, particularly costs for reaching OOS girls.	Cohort study (Retrospective)	Zimbabwe	Primary and secondary data	10‐14 years. 1,569,905 doses administered in 2 years	GAVI, WHO, Zimbabwe Ministry of Health and Child Care	FC per dose: $0.53 EC per dose: $1.31 **2025 US$ (inflation‐adjusted): FC/dose: $0.66; EC/dose: $1.63** *Reference year: 2020, Assumed*	Combined district and health facility total costs: FC US$828,731; EC US$2,060,943.	The study recommends that to ensure equitable access to HPV vaccination, future studies should assess the costs and effectiveness of various strategies to reach OOS girls.
5.	Hsiao et al., (2023) [50]	To evaluate the financial and economic costs of the national rollout of an HPV vaccination program in school‐aged girls in sub‐Saharan Africa and the potential costs associated with a single‐dose HPV vaccine program.	Mixed Methods Study	Tanzania	Primary and secondary	School‐aged girls in Tanzania	The DoRIS, Bill and Melinda Gates Foundation.	Financial cost: $5.17 per 2 dose and $2.51 per dose. Economic cost: $22.34 per 2 doses and $12.18 per dose. **2025 US$ (inflation‐adjusted): FC: $6.14 per 2‐dose and $2.98 per dose. EC: $26.54 per 2‐dose and $14.47 per dose** *Reference year: 2021, Assumed*	One‐ vs two‐dose scenarios: Total financial and economic costs were US$10,117,455 and US$45,683,204, respectively, at a financial cost of $5.17 per two‐dose FIG, and an economic cost of $23.34 per FIG. In a one‐dose scenario, the cost per FIG reduced to $2.51 (financial) and $12	The findings may help other countries yet to include HPV vaccines in their routine immunization to understand the need to prioritize funding for HPV vaccine
6.	Mwenda et al., (2023) [48]	To assess the potential impact, cost‐effectiveness, and budget impact of GARDASIL‐4 and alternative products in Kenya.	Quantitative Descriptive Study	Kenya	Primary and secondary	10‐year‐old girls, 14 birth cohorts between 2007 − 2020	GAVI, Government of Kenya, PATH	Cecolin: $3:00 per dose Cervarix: $5.18 per dose Gardasil‐4: $4.50 per dose Gardasil‐9: $25 per dose **2025 US$ (inflation‐adjusted): Cecolin: $3.56/dose; Cervarix: $6.15/dose; Gardasil‐4: $5.35/dose; Gardasil‐9: $29.70/dose** *Reference year: 2021, Assumed*	Cost‐effectiveness: HPV vaccination could reduce CC burden by 42–60%. A single‐dose strategy would be cost‐saving compared to no vaccination for all three vaccines currently supported by Gavi.	As Kenya graduates from Gavi support, significant government funding is required to reach and sustain HPV vaccine coverage targets
7.	Mvundura et al., (2024) [47]	To estimate the ongoing financial and economic costs and operational context of HPV vaccine delivery in three regions of Ethiopia (Addis Ababa, Afar, and Amhara).	Mixed Methods Study	Ethiopia	Primary and secondary	60 health facilities, 17 wards/districts, and 9 zones/sub‐cities	Bill and Melinda Gates Foundation	Financial Cost: $2.23 Economic Cost: $7.19 **2025 US$ (inflation‐adjusted): FC: $2.45; EC: $7.91** *Reference year: 2022, Assumed*	Regional variations in the estimates: the mean financial cost per dose was between $1.17 to $7.18. Mean economic cost per dose ranged from $5.80 to $18.13 across the three regions.	The study reveals regional variations in HPV vaccine costs, which stakeholders need to account for when making funding allocation decisions.

### 3.2 Year of Publication

**Figure 2:**
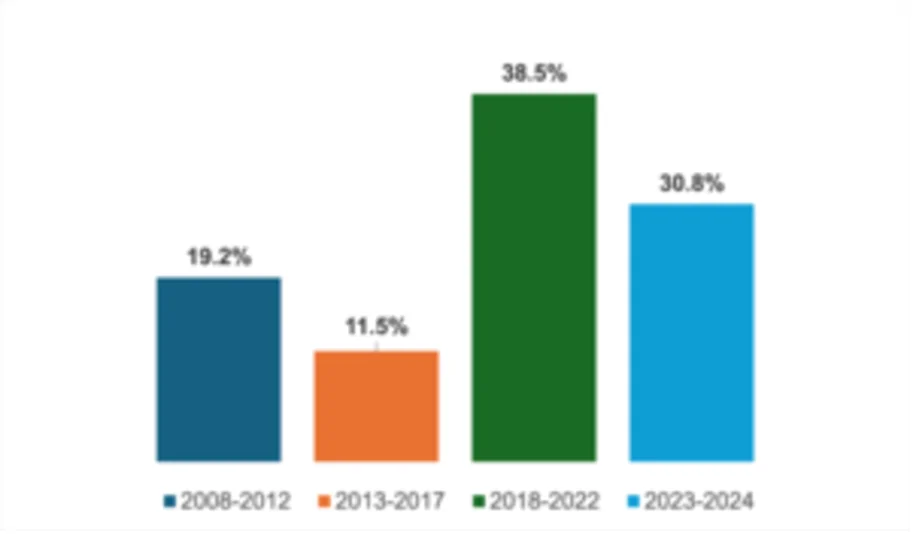
Distribution of studies by year of publication.

Figure 2 illustrates the frequency distribution of studies on HPV financing across four time periods. We observed some variations in research activity over the years as the highest percentage of studies (38.5%) was conducted between 2018 and 2022, during which research interest in the subject peaked. The lowest research output (11.5%) was recorded between 2013 and 2017, which indicated a decline compared to 2008–2012 (19.2%). However, research activity surged after 2017, followed by a slight decrease in the 2023–2024 period (29.6%). Notwithstanding the decline, there has been growing attention on the subject, particularly in recent years.

### 3.3 Study Designs

**Figure 3:**
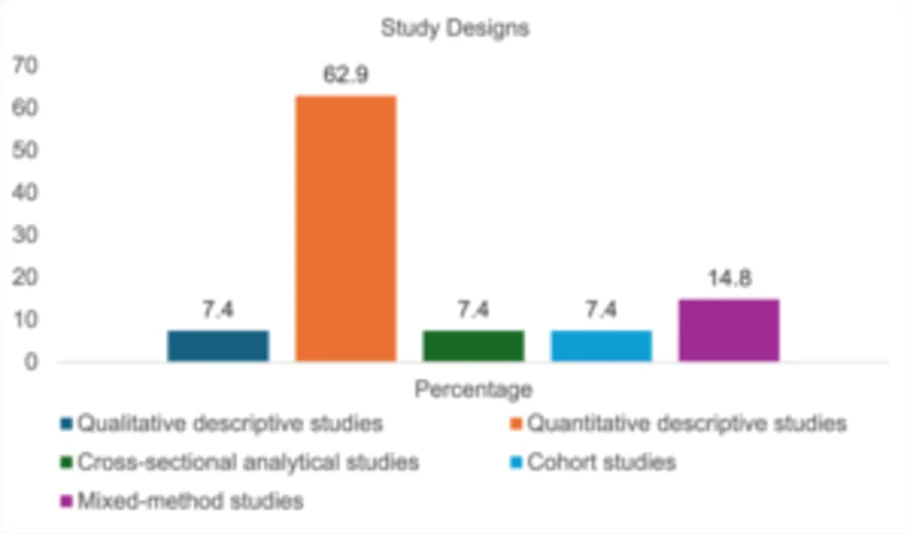
Distribution of studies on HPV financing by study design.

The studies included in the review employed a variety of research designs, falling into four main categories which are qualitative studies (*n* = 2; 7.4%) [46, 48], quantitative non‐randomized Studies (*n* = 4; 14.8%) [30, 45, 47, 49]; Quantitative descriptive studies (*n* = 17; 62.9%) [17, 33, 34, 35, 37, 38, 39, 40, 41, 42, 43, 49, 50, 52, 53] and Mixed‐method Studies (*n* = 4; 14.8%) [29, 42, 51, 54]. Among the quantitative non‐randomized studies, two studies were retrospective cohort studies [30, 49] and the other two were cross‐sectional analytical studies [45, 47] (Figure 3).

### 3.4 Costs and Purchasing Power Across the Study Period

**Figure 4:**
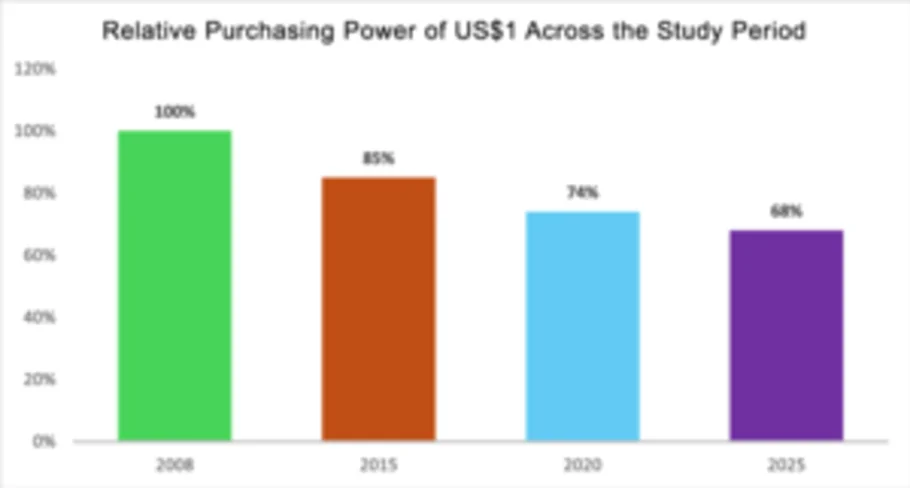
Relative Purchasing Power of US$1 Across the Study Period (2008–2025). *Source:* U.S. Bureau of Labor Statistics, CPI‐U annual averages [25].

To aid the interpretation of findings related to costs, it is important to provide background information about the purchasing power of United States Dollar (USD) over the years. Figure 4 illustrates the relative purchasing power of US$1 across the study years (2008–2025). To preserve the integrity of the original studies, HPV‐related cost estimates were reported exactly as presented in the respective articles and directly adjusted to a common currency year (2025) (see Tables 1A–E, Figure 4 and Supporting File 2 for the adjusted costs). From 2010 to 2024, the U.S. dollar steadily lost purchasing power, though the pace varied considerably. Between 2010 and 2020, losses were modest—typically 0.5% to 1.5% per year — reflecting the low‐inflation environment of that decade. The dollar lost roughly 16% cumulatively over those ten years. The real damage came post‐2020: inflation surged dramatically in 2021 (−2.2%) and 2022 (−4.0%), the steepest single‐year losses in the entire period, driven by pandemic‐era supply disruptions and stimulus spending. By 2024, the dollar had lost nearly 32% of its 2008 value, with more than half that erosion occurring in just 4 years (2021–2024).

Figure 4 presents the relative purchasing power of the United States Dollar (US$), using 2008 as the reference year (US$1 = 100%). Based on an estimated average annual decline of 2.2% in purchasing power, US$1 in 2008 was approximately equivalent to US$0.68 in 2025, representing an overall 32% decline in real value over the 17‐year period. Consequently, an item costing US$1 in 2008 would require approximately US$1.48 in 2025 to maintain equivalent purchasing capacity. The figure therefore provides contextual insight into inflation‐related changes in the real value of money over time and supports more informed interpretation of HPV vaccine cost estimates reported across different study years without altering the original reported values.

### 3.5 Quality Appraisal Outcomes

The quality of all 27 included studies was assessed as above average with appraisal scores ranging between 4/7 and 7/7 (Tables S5–S5 [Supplementary file]). The lowest score of 4/7 was obtained by a quantitative descriptive study [32]. Conversely, the highest score of 7/7 was achieved by five studies, including the qualitative descriptive studies [46, 48], the quantitative descriptive study by Simuyemba et al. [34], and two mixed‐method studies [42, 51]. The four quantitative non‐randomized studies scored between 4.5/7 [47] and 6/7 [30, 49]. The remaining 16 quantitative descriptive studies were scored between 5/7 [44] and 6/7 [17, 33, 35, 37, 38, 39, 40, 41, 43, 50, 52, 53] whereas the two remaining mixed‐method studies scored between 5.5/7 [54] and 6.5/7 [29], maintaining an overall “above average” rating.

### 3.6 Country of Study

Single‐country studies constituted more than 2/3rd of the articles (76.0%), cutting across different subregions of Africa, including Eastern Africa [32, 33, 34, 41, 43, 50, 51, 52, 53, 54], Western Africa [30, 39, 44, 45, 46, 47], Southern Africa [29, 31, 49], and Northern Africa [37, 40].

Additionally, there are multi‐country research works on the subject traversing at least 2 subregions of Africa such as Eastern and Southern Africa [35, 38], Eastern and Western Africa [17], East, West and North Africa [42], two reported HPV financing globally including countries in Africa [36, 48].

### 3.7 Sources of Funding and Technical Support for HPV Vaccine

To fund HPV vaccination, the majority of the countries assessed (or relied on) donations from the vaccine manufacturer GAVI (68%), either solely or with support from other funders such as the Bill and Melinda Gates Foundation. The technical partners including the United States Centers for Disease Control and Prevention, World Health Organization (WHO), The DoRIS, National cancer institute, and PATH, provided non‐financial assistance such as policy guidance, surveillance, training, and implementation support. In some countries, the Ministry of Health supplemented GAVI donations with domestic resources. Some countries also received funding/donations from sources other than GAVI (WHO, Bill and Melinda Gates Foundation, PATH, Pan American Health Organization (PAHO) Revolving Fund).

### 3.8 Economic and Financial Cost of HPV Vaccine

Across Africa, the implementation of the HPV vaccination program and associated costs were country‐specific. Many studies contextualized HPV vaccine costs by comparing the economic and financial costs. In line with WHO Cervical Cancer Prevention and Control Costing (C4P) framework [55], economic cost (EC) is described as costs comprising financial expenses comprising both delivery and vaccine costs, along with the utilization of existing resources within the health system such as staff time equipment and facility use. While financial cost (FC) is the cost expended on resources meant to implement the program [29] or direct expenditures for vaccine delivery [55], excluding vaccine procurement unless otherwise specified. The studies reported a wide range of EC and FC for HPV vaccination across different countries, demonstrating the EC to be higher than the FC. However, it is important to note that all costs have been standardized to USD.

The average EC per dose and fully‐immunised girl (FIG) was US$17.59 and US$52.29, respectively for a 3‐dose HPV vaccine schedule in Mozambique. The FC was however much lower at US$6.07 per dose and US$17.95 per FIG. When the doses/personnel were reduced, EC per dose and FIG declined to US$15.53 and US$31.14 each [29]. This is comparatively lower than the estimates for Zimbabwe where the mean FC per dose was $19.76, and the EC was $45.00, while per FIG, the FC and EC were US$ 40.03 and US$ 91.19 each [31]. In Zimbabwe, FC are direct expenditures by government and donors for HPV vaccine introduction activities. EC are described as FC plus opportunity costs, such as time for existing personnel and in‐kind resources [31]. Cost per FIG in Uganda was US$21 [48]. Zambia had a lower FC at US$6.0 per dose and US$11.9 per FIG, while the EC was US$23.0 per dose and US$46.0 per FIG [34]. The higher FC per FIG in Mozambique compared to others was attributed to a high percentage of out‐of‐school girls (OOG), which reduced vaccine coverage and, therefore, increased the cost per FIG [29].

Excluding the vaccine cost, the FC to fully immunize a girl in Tanzania was higher (US$5.7) [32] than that of Ethiopia as the FC per dose averaged $2.23. The EC (including opportunity costs) was $7.19 [17].

The overall or total FC and EC for HPV vaccine in Zimbabwe were US$ 229 144 and US$ 521 946, respectively [31]. A recent study in the country estimated the overall FC and EC (district and health facility level) of HPV vaccination to be higher at a rate of US$828,731 and US$2,060,943, respectively [49].

Similarly, in a multi‐country study, the mean annual FC per health facility was lower (Ethiopia‐ $421, Rwanda‐ $218, Senegal‐ $497, and Uganda‐ $423) than EC (Ethiopia‐ $1,550, Rwanda‐ $1,082, Senegal‐ $2,169, and Uganda‐ $1,212). Likewise, the FC per dose ranged from $0.27 to $3.32, while the EC per dose ranged from $3.09 to $11.94 [42]. Conversely, the estimated annual cost of Burkina Faso vaccination program varied significantly depending on the vaccine used: CECOLIN® (US$2.9 million), GARDASIL‐4® (US$4.1 million), CERVARIX® (US$4.4 million), GARDASIL‐9®(US$19.8 million) [39].

In addition to country‐level differences, some in‐country variations were also reported. Such as regional variations in service delivery, costs, and implementation strategies for HPV vaccine delivery in three regions of Ethiopia. Afar region had the highest cost per dose (US$7.18 financial, US$18.13 economic) due to lower vaccination volumes (Addis Ababa, Afar, and Amhara) [17].

### 3.9 Cost of HPV Vaccination Program

The total FC estimated for 5 years in Tanzania was US$18.3 million while EC was US$47.3 million. The cost per FIG with the HPV vaccine is $6.68 (financial) and $17.31 (economic) [50]. Likewise, the total FC of Tanzania's national HPV vaccination program was $10.1 million, while the EC (including opportunity costs) was $45.7 million [54]. A recent study in Tanzania estimated the total economic project costs for delivering 3 doses of HPV vaccine (at US$5 per dose) to 4,211 girls at US$349,400 [43].

The total cost of HPV program in Kenya using a single‐dose vaccine is projected to be US$ 43222 million, over 10 years (2020‐2029) [52]. The estimated annual program cost could exceed $10 million if Kenya achieves its 90% coverage target and graduates from Gavi support, requiring significant government funding. A single‐dose strategy was found to be cost‐saving, offering similar benefits at a lower cost [52]. This is however lower than the projected annual program cost for Ghana ($11.2 million to $15.4 million), which is 11%–15% of Ghana's immunization budget [44].

At an assumed vaccine price of US$5 per dose, the estimated cost of establishing an HPV vaccine program in Tanzania over 5 years (2011–2015) was US$9.2 million (excluding vaccine costs) and US$31.5 million (including vaccine costs) [32]. This is lower than the projected cost in Morrocco, where an assumed vaccine price of US$10 per dose, and a 70% coverage rate would amount to US$1,150 per YLS [40]. In Kenya, the discounted program cost varied for different vaccine brands‐CECOLIN, CERVARIX, GARDASIL4, and GARDASIL‐9. This was estimated at US$ 74, US$ 90, US$ 86, and US$ 380 million each [52].

The largest expenses were vaccine procurement [50, 54] and injection supplies (46% of FC, 55% of EC), followed by service delivery (38% of FC) [54]. Others are personnel, building overhead and vehicles, microplanning, supplies and service delivery and outreach [34].

It is important to note that the costs reported in the current study are the values presented in each study and correspond to the reference years stated by the respective authors. Because the reviewed studies used different base years and currencies, direct cost comparisons should be interpreted with caution (see Figure 4 for value of USD1 across the study years). Hence, our analysis shows relative differences and trends across included studies rather than standardized monetary value. Differences in costing scope, vaccination schedules (single‐dose vs. multi‐dose), delivery strategies (school‐based, facility‐based, or outreach), and country‐specific implementation contexts contributed to variations in reported costs across studies. To improve comparability, all monetary values were interpreted in relation to the relative purchasing power of the US dollar across study years.

### 3.10 Disaggregation of Economic and Financial Costs

Service delivery constituted a significant proportion of both EC and FC costs of HPV vaccine across Africa. This was the case in Rwanda where service delivery constituted the major cost unit for HPV vaccine (Gardasil) on account of outreach allowances paid to staff [33]. Equally in Senegal, training (42%) and service delivery (30%) were the biggest FC, while service delivery (57%) was also the biggest EC followed by personnel time [30]. In Zimbabwe, training (37%) and service delivery (30%) took the major portion of the total FC. For the EC, service delivery consumed almost half (45%) of the total, followed by training (19%). The total service delivery cost per dose was US$0.16 (FC) and US$0.59 (EC) [49], this was a sharp decline from the estimate in a previous study where the lowest service delivery costs per dose ($1.97 financial, $6.79 economic) occurred when overlapping groups of girls were being vaccinated [31]. However, efforts to reach OOS girls increased FC by US$2.99 per dose and EC by US$7.79 per dose [49].

The cost of vaccine delivery was higher for a two‐dose ($12.94 per individual) than a single‐dose ($6.47 per individual) vaccine in a multi‐site study. Both FC and EC also declined drastically across the African countries examined [17]. In Tanzania, social mobilization, information, education, and communication (IEC) are the major costs of HPV vaccination. Next to this are service delivery costs [32]. Similarly, in Zimbabwe, social mobilization and information materials took a major proportion of the FC (24.1%), while service delivery consumed EC the most at 21.7% [31].

In a multi‐country study, opportunity costs (personnel time for service delivery) made up the largest share of expenses across the countries assessed (Ethiopia‐65%, Rwanda‐57%, Senegal‐62%, Uganda‐44%) [42]. In Ethiopia, costs varied across health system levels, however, service delivery was the largest financial expenditure and human resource time contributed considerably to EC [17].

### 3.11 Investment and Recurrent Costs of HPV Vaccine

Also reported in some of the studies were investment and recurrent costs. Investment costs (IC) represent costs that are expected to last longer than 1 year such as planning, training, social mobilization, cold chain (supplement), and other program activities. Recurrent costs (RC) are periodic costs such as service delivery, monitoring, supervision, cold chain (recurrent), among other frequent activities [30].

The investment cost of HPV vaccine was higher than the recurrent cost in countries like Senegal where IC and financial RC per dose of HPV vaccine was $1.85 and $1.22, respectively [30]. When compared to the cost of other vaccines, HPV vaccine consumed the highest RC in Rwanda. However, its annual cost was lower due to the low cost of procurement [33].

### 3.12 Willingness to Pay for HPV Vaccine

Just 2 studies examined private individuals’ willingness to pay for HPV vaccine. In Nigeria, 91.6% of mothers were willing to pay for HPV vaccination for their daughters. The average willingness to pay was $11.68 per fully vaccinated girl. However, this was lower than the estimated cost of vaccination at the Gavi‐subsidized price ($18.16 to $19.26), and the lowest public‐sector price ($35.16 to $36.26) [47]. A similar study found that over 58% of men were willing to pay for vaccination. The willingness to encourage and pay for vaccination was significantly influenced by residency, education, marital status, and income [45]. However, HPV vaccination funded through out‐of‐pocket payments is believed to make access to vaccines inequitable. Hence, a publicly funded HPV vaccination program patterned after the COVID‐19 vaccination roll‐out was proposed in Ghana [46].

### 3.13 Cost and Mode of HPV Vaccine Delivery

Mode of HPV vaccine delivery was a major determinant of the cost. In Tanzania, the health facility‐based vaccination method ($3.51) was estimated to be cheaper than the school‐based method ($4.78) due to transportation and outreach costs [32]. In contrast, school‐based vaccination was the most cost‐effective strategy in Zimbabwe and Zambia, while health facility and outreach strategies were significantly more expensive. In Zimbabwe, the FC per FIG was $5.34 in schools, $34.90 in health facilities, and $288.63 in outreach, while the EC was $17.39, $41.25, and $635.84, respectively [31]. Similarly, in Zambia, school‐based vaccine delivery was the cheapest with EC at USD13.2 per dose and USD 26.4 per FIG, whereas, outreach costs USD118.8 per dose and USD237.7 per FIG. At the health facilities, the cost was USD365.2 and USD730.4 per dose and FIG, respectively [34].

Per FIG, costs were lower when a class‐based delivery strategy was utilized rather than age‐based delivery. Incremental economic scaled‐up costs for class‐based vaccination of 50,290 girls were estimated at US$1.3 million. The economic scaled‐up costs per FIG were US$26.41 (vaccine cost inclusive). The incremental economic cost when vaccine costs are excluded was US$3.09 per dose and US$9.76 per fully‐immunized girl. Financial scaled‐up costs, (vaccine costs and salaries excluded), were estimated at US$1.73 per dose [43].

In a two‐dose scenario, the FC per FIG was $5.17, and the EC was $23.34 in Tanzania. Adopting a one‐dose vaccine schedule is projected to significantly lessen costs, with the FC per FIG falling to $2.51 and the EC to $12.18 [54]. This is similar to findings in Burkina Faso where a single‐dose vaccination strategy reduced costs and improved cost‐effectiveness by more than half [39]. As observed in Tanzania, the one‐dose strategy would lower costs by reducing vaccine procurement, injection supply, and service delivery costs while also addressing loss to follow‐up issues [54].

### 3.14 Impact and Cost‐Effectiveness of HPV Vaccine

The economic impact and cost‐effectiveness of HPV vaccine were assessed by comparing different vaccine brands, dosages, efficacy in preventing cervical cancer (CC), and national economic factors.

In Burkina Faso, CECOLIN was the most cost‐effective vaccine, both with and without cross‐protection [39], while in Kenya, CECOLIN was most cost‐effective without cross‐protection, and CERVARIX provided better health benefits when cross‐protection was considered [52]. The 9‐valent vaccine was highly cost‐effective in Kenya if the added cost per vaccinated girl was ≤ $9.7 and cost‐effective up to $27.3 under a higher willingness‐to‐pay threshold, while in Uganda, cost‐effectiveness was lower at $8.3 and $23.4, respectively [38]. In Tunisia, adding HPV vaccination to cervical cancer (CC) prevention was financially sustainable, with an incremental cost‐effectiveness ratio (ICER) of $1,920.8 per life‐year saved and $2239.3 per quality‐adjusted life‐year [37].

Ghana's most cost‐effective strategy was routine vaccination of 9‐year‐old girls with a one‐time campaign for 10–14‐year‐olds using the bivalent vaccine ($158 per DALY averted) [44]. A shift from a two‐dose to a single‐dose schedule could cut costs by 45%–55% in Ethiopia, Kenya, and Uganda, saving $33.5 M, $8.2 M, and $4.7 M over ten years [17]. In Ethiopia, routine two‐dose HPV vaccination at 50% coverage could prevent 970,000 CC cases and 932,000 deaths over a century while averting 33,900 catastrophic health expenditures [53]. In Uganda, a one‐dose strategy reduced CC cases by 15%–16% over 40 years, while a two‐dose strategy reduced cases by 21% but required double the investment [41].

In Eastern Africa (Kenya, Mozambique, Tanzania, Uganda, and Zimbabwe), HPV vaccination alone reduces lifetime CC risk by 36–45%, and adding one HPV DNA screening at age 35 further reduces risk to 43%–51%, with cost‐effectiveness favourable at ≤ $10 per vaccinated girl [35]. A study across 36 African countries found 9–17 deaths prevented per 1,000 vaccinated, though affordability remained a concern, as increasing the per‐vaccinated‐girl cost from $10 to $25 raised total FC from $900 M to $2.25B [36]. In Morocco, HPV vaccination at 70% coverage reduces CC risk by 62% at $1,150 per YLS, while combining vaccination with screening improves risk reduction to 69% but at a higher cost of $2,843 per YLS [40]. Cost‐effectiveness in Kenya and Uganda was also influenced by multiple HPV infections, with incremental cost per dose needing to stay below $3.2 in Kenya and $2.8 in Uganda at a willingness‐to‐pay threshold of 1x GDP per capita [38].

### 3.15 Challenges With HPV Financing in Africa

Vaccine affordability and analysis of the costs of delivering HPV vaccine are key considerations in many of the reviewed studies. However, HPV vaccine financing in Africa faces several challenges, primarily due to reliance on external funding sources such as Gavi, which raises concerns about long‐term sustainability once such support diminishes [34]. Although Gavi subsidizes a significant portion of vaccine costs in Africa, countries like Zambia still have co‐financing obligations, making financial sustainability a critical issue [34]. Additionally, the lack of locally generated evidence for financial planning has led to decision‐making being influenced more by global funding priorities rather than country‐specific data [34].

Another major challenge is the high delivery cost of HPV vaccines, which varies widely between countries depending on factors such as delivery strategies, target populations, integration into existing health services, and operational contexts [42]. For instance, Morocco faces uncertainty in vaccine pricing since it is ineligible for Gavi support [40]. Similarly, in Burkina Faso, securing funds for HPV vaccination is inhibited by the high vaccine cost and the country's economic problems, which makes financial sustainability difficult without Gavi support [39]. Tunisia also struggles with securing funds due to high vaccine prices [37].

Reaching OOG was another financial burden in Mozambique, where the higher cost of vaccinating OOG poses challenges for program scalability and sustainability [29]. In both Kenya and Uganda, given limited national resources, experts face the dilemma of demonstrating lower‐cost strategy and affordability of the HPV vaccine compared to other competing healthcare needs [38].

## 4 Discussion

Database search generated 326 articles at initial search out of which 61 duplicates were removed, thus producing 265 unique articles. After assessing titles and abstracts for relevance, 27 articles met the inclusion criteria and were selected for the final analysis. The included studies are very heterogeneous in terms of the year of publication, study design, methodology, analysis, and findings. The reviewed studies indicate that the economic cost (EC) of HPV vaccination is generally higher than the financial cost (FC) across various countries. However, comparing costs across countries proved challenging due to the wide variation in reported EC and FC, which included per‐dose costs, costs per FIG, and total program expenditures. The FC per dose varies from $0.27 to $19.76, while the EC per dose ranges from $3.09 to $45.00. Similarly, the FC per FIG ranges from $6.07 to $40.03, whereas the EC per FIG is between $17.31 and $91.19. Although HPV costs differ across the globe, the costs in the current review are higher but within the range that has been reported for some locations such as Sri Lanka [56] and the Philippines [57]. Country‐level and regional variations in the cost of HPV vaccine reflect dissimilarities in vaccine procurement, service delivery [30], implementation approaches [49], and efficiency of the healthcare system [57].

Service delivery cost is a major driver of HPV vaccination influencing both FC and EC across Africa [33, 34, 51], and Asia [57]. This was followed by the cost of social mobilization and IEC [31, 32]. These indicate that human resource costs play a significant role in HPV vaccination programs depending on the service delivery strategies utilized. HPV vaccination is widely reported to be cost‐effective across Africa, with variations based on vaccine type/brand, dosing schedule, CC prevention, and national economic factors [17, 37, 39, 42, 44, 53], however vaccine affordability is a major concern. Evidence from the current review indicates that investing in HPV vaccination could prevent CC‐related morbidity and mortality, remarkably in high‐burden countries like Eswatini, Malawi, Zambia, and Uganda [58]. Also, evidence reveals that a combination of HPV vaccination with CC screening can further enhance protection and cost‐effectiveness [35, 40, 50]. This supports previous studies on the cost‐effectiveness of HPV vaccine [59, 60].

The evidence synthesized in this review suggests that transitioning to a single‐dose schedule can significantly reduce procurement, delivery, and cold chain costs while cost‐effectiveness is safeguarded. Moreover, shifting to a one‐dose vaccine schedule reduced the FC and EC in Tanzania [54] and Burkina Faso [39]. In addition to cost reduction, the findings suggest that a single‐dose vaccine schedule helped to maintain effectiveness, reduce loss to follow‐up and other logistical burdens on health systems. Single‐dose HPV vaccine was found in other studies to be very efficacious in preventing CC similar to multiple doses [61, 62, 63, 64]. The Joint Committee on Vaccination and Immunization (JCVI) also affirms the efficacy of single‐dose vaccines [65].

Considering that cost is a major barrier to HPV vaccine introduction and coverage in many low‐resource countries [66, 67], governments need to shift to cheaper alternatives and allocate sufficient funds for HPV vaccination programs, especially as some countries transition from Gavi support, which could put more strain on limited resources. African countries need to prioritize vaccine brands that are cost‐effective to maximize health benefits and minimize costs. Weighing the benefits of cross‐protection is also important to ensure long‐term outcomes [38, 41, 63].

The current review also suggests the need to adopt targeted strategies to reach out‐of‐school girls and other populations experiencing low vaccine coverage [29, 34]. In addition, strengthening health systems and human resources is crucial to maintaining effective vaccine delivery and monitoring programs. The current review shows that the mode of delivery impacts the cost of HPV vaccine, such as health facility‐based versus school‐based, and class‐based versus age‐based strategy. School‐based delivery is more cost‐effective in Zimbabwe [31], and Zambia [34], while health facility‐based vaccination is cheaper in Tanzania [32]. This implies that countries should tailor HPV vaccine delivery strategies to their specific context.

While integration into routine facility‐based immunization is a theoretically sound goal for sustainability, its success is contingent upon a major behavioral shift among caregivers and a redesign of service delivery. In contexts where adolescent health‐seeking at facilities is not the norm, this model faces significant hurdles, including transportation costs, caregiver time constraints, and a lack of perceived need for preventive care for healthy adolescents [68, 69]. Lessons from countries that have attempted facility‐based integration highlight the critical need for parallel investments in social mobilization and community demand generation. For instance, experiences in Malawi and Tanzania demonstrate that without a comprehensive communication strategy to educate families, facility‐based uptake can remain low [70, 71]. A successful transition will likely require a multi‐year strategy that combines strengthening facility readiness with robust community outreach, potentially using school‐based delivery as a foundational step to build coverage and awareness before a gradual shift to a fully integrated model [72].

The transition to a facility‐based model is less a vaccine delivery tactic and more a health system transformation. It might require a 2–3‐year horizon and a significant upfront investment in social and system enablers, not just vaccines [73]. The business case is not immediate cost‐saving but is built on achieving sustainable, equitable coverage and creating a durable platform for adolescent health [74]. Attempting this shift without allocating the necessary time and budget for social mobilization and health worker training will almost certainly lead to a sharp decline in vaccination coverage, wasting previous investments and leaving a generation of girls unprotected [70].

Lastly, studies on willingness to pay for HPV vaccine were few, evidence points to a positive attitude toward paying out‐of‐pocket for the vaccine at a certain cost threshold which is lower than the subsidized rate [47]. Factors such as income, education, residency, and marital status were associated with willingness to pay [38]. This is similar to findings among health workers in China [75]. It however contradicts that among women living with HIV where willingness to pay for HPV vaccine was low due to inadequate knowledge [76].

HPV vaccine financing in Africa faces major sustainability challenges due to fiscal dependence on Gavi, high and variable delivery costs, and difficulties securing funds for non‐Gavi eligible countries. This leads to financial burdens, competing healthcare priorities, and decision‐making influenced by global rather than local evidence. The findings underscore a precarious reality for HPV vaccination programs across Africa, where financial sustainability is threatened by a triad of interconnected challenges. The dependence on external funding, particularly from Gavi, creates a fundamental vulnerability. While donor support is crucial for initiation, long‐term dependency without a clear transition plan jeopardizes program continuity when support diminishes [77]. This is exacerbated for non‐Gavi eligible countries, which face prohibitive vaccine prices without a safety net, forcing them into difficult budgetary negotiations against other pressing health priorities [78, 79].

The high and variable delivery costs further complicate financial planning. The significant variation in cost per dose reported across countries reflects differences in delivery strategies and operational contexts, making it difficult to establish standardized budgeting tools [18]. This variability, combined with the high cost of reaching out‐of‐school girls, means that the true financial burden is often underestimated, leading to funding shortfalls that can stall scale‐up. Ultimately, this financial instability skews decision‐making paradigms. The pressure to align with global funding streams can inadvertently sideline country‐specific data and needs, a phenomenon noted in other global health initiatives [80]. When national decisions are influenced more by the availability of external funds than by locally generated evidence on lower‐cost strategy and burden of disease, programs risk being misaligned with national health priorities, undermining their political legitimacy and long‐term integration into domestic health systems.

## 5 Conclusion

This scoping review has synthesized current evidence on the financing of HPV vaccination in Africa, revealing significant variations in costs, funding sources, and delivery models. Donor dependence—particularly on Gavi—remains central to vaccine procurement and program implementation. However, as many countries prepare to transition from donor aid, there is an urgent need for sustainable domestic financing strategies in the long term. It is immediately actionable to transit to single‐dose HPV vaccination schedule which significantly reduces both financial and logistical burdens. Service delivery costs, including personnel and outreach, account for the largest share of expenditures, while school‐based and single‐dose delivery models emerge as cost‐saving approaches. Despite evidence of cost‐effectiveness, affordability and access—especially for out‐of‐school girls—remain key challenges. Moving forward, governments must prioritize HPV vaccine financing within national budgets, adopt economically feasible procurement practices, and tailor delivery strategies to local contexts. Strengthening health systems and investing in long‐term solutions will be essential to achieving the WHO 90‐70‐90 elimination targets and reducing the burden of cervical cancer across the continent.

## Author Contributions

**Kafayat Aminu:** conceptualization, data curation, formal analysis, funding acquisition, investigation, methodology, visualization, project administration, resources, software, supervision, validation, writing – original draft, writing – review and editing. **Jimoh Amzat:** conceptualization, methodology, visualization project administration, resources, validation, writing – original draft, writing – review and editing. **Rita Amarachi Nwebo:** conceptualization, data curation, investigation, methodology, project administration, resources, software, validation, visualization, writing – original draft. **Precious Chika Nnannah:** conceptualization, data curation, investigation, methodology, project administration, resources, software, validation, visualization, writing – original draft. **Chiamaka Norah Ezeagu:** resources, writing – original draft. **Aboyowa Arayuwa Edukugho:** writing – review and editing. **Olubukola Omobowale:** resources, writing – review and editing. **Abba Shehu:** writing – review and editing. **Yovanthi Anurangi Jayasinghe:** resources, writing – review and editing. **Ganiyat Amzat:** methodology, visualization, writing – original draft, writing – review and editing. **Kehinde Kazeem Kanmodi:** conceptualization, data curation, funding acquisition, investigation, methodology, visualization, project administration, resources, software, supervision, validation, writing – original draft, writing – review and editing.

## Funding

This study was self‐funded.

## Ethics Statement

Not applicable. This study did not collect data from human or animal subjects but an open research repository.

## Conflicts of Interest

Kehinde Kazeem Kanmodi is an Editorial Board member of *Health Science Reports* and co‐authors of this article. To minimize bias, he was excluded from all editorial decision‐making related to the acceptance of this article for publication. Other authors have no conflict of interest to declare.

## Transparency Statement

All authors have read and approved the final version of the manuscript. The corresponding author and manuscript guarantor, Kehinde Kazeem Kanmodi, affirms that this manuscript is an honest, accurate, and transparent account of the study being reported; that no important aspects of the study have been omitted; and that any discrepancies from the study as planned (and, if relevant, registered) have been explained. Also, Kehinde Kazeem Kanmodi (the corresponding author and manuscript guarantor) had full access to all of the data in this study and takes complete responsibility for the integrity of the data and the accuracy of the data analysis.

## Supporting information


Supporting File 1

Supporting File 2

## Data Availability

The authors confirm that the data supporting the findings of this study are available within the article and its supplementary materials.
