# Supplementary material for: Financing of Human Papillomavirus Vaccination in Africa: A Scoping Review
Source: Health Sci Rep. 2026 Sep 25;9(10):e73254. doi: 10.1002/hsr2.73254 (PMC13614079; doi:10.1002/hsr2.73254)
Supplement: Supplementary file 1 [file HSR2-9-e73254-s001.docx]

**Supplementary File 2**

**Table S10. Inflation-adjusted HPV vaccine cost estimates across included studies, standardized to 2025 US$**

*All cost estimates extracted from the 27 included studies (Tables 1A–1E) are already denominated in US$ in the original source. To enable comparison across studies conducted in different years, each estimate was additionally re-expressed in a common reference year (2025 US$) using the US Bureau of Labor Statistics Consumer Price Index for All Urban Consumers (CPI-U), following the general approach to cost-year standardization used in health-economics systematic reviews.a Both the originally reported value and the inflation-adjusted value are presented so that the primary data are preserved alongside the standardized estimate.*

| **S/n** | **Author(s), year (Ref #)** | **Country** | **Cost item** | **Cost type** | **Reported cost (original US$)** | **Reference / cost yeara** | **Basisb** | **CPI-U deflatorc** | **Inflation-adjusted cost (2025 US$)d** |
| --- | --- | --- | --- | --- | --- | --- | --- | --- | --- |
| 1 | Alonso et al., 2019 (29) | Mozambique | Per dose | Financial cost | $6.07 | 2014 | Stated | 1.360 | **$8.25** |
| 2 | Alonso et al., 2019 (29) | Mozambique | Per dose | Economic cost | $17.59 | 2014 | Stated | 1.360 | **$23.92** |
| 3 | Alonso et al., 2019 (29) | Mozambique | Per FIG | Financial cost | $17.95 | 2014 | Stated | 1.360 | **$24.41** |
| 4 | Alonso et al., 2019 (29) | Mozambique | Per FIG | Economic cost | $52.29 | 2014 | Stated | 1.360 | **$71.11** |
| 5 | Brennan et al., 2022 (30) | Senegal | Per dose | Financial cost | $3.07 | 2020 | *Assumed* | 1.244 | **$3.82** |
| 6 | Brennan et al., 2022 (30) | Senegal | Per dose | Economic cost | $7.56 | 2020 | *Assumed* | 1.244 | **$9.40** |
| 7 | Hidle et al., 2018 (31) | Zimbabwe | Per dose | Financial cost | $19.76 | 2016 | *Assumed* | 1.341 | **$26.50** |
| 8 | Hidle et al., 2018 (31) | Zimbabwe | Per dose | Economic cost | $45.00 | 2016 | *Assumed* | 1.341 | **$60.36** |
| 9 | Hidle et al., 2018 (31) | Zimbabwe | Per FIG | Financial cost | $40.03 | 2016 | *Assumed* | 1.341 | **$53.69** |
| 10 | Hidle et al., 2018 (31) | Zimbabwe | Per FIG | Economic cost | $91.19 | 2016 | *Assumed* | 1.341 | **$122.31** |
| 11 | Hutubessy et al., 2012 (32) | Tanzania | Per dose | Financial cost | $10.77 | 2011 | Stated | 1.431 | **$15.42** |
| 12 | Hutubessy et al., 2012 (32) | Tanzania | Per dose | Economic cost | $12.24 | 2011 | Stated | 1.431 | **$17.52** |
| 13 | Ngabo et al., 2015 (33) | Rwanda | Per dose | Financial cost | $10.23 | 2013 | *Assumed* | 1.382 | **$14.13** |
| 14 | Ngabo et al., 2015 (33) | Rwanda | Per dose | Economic cost | $14.45 | 2013 | *Assumed* | 1.382 | **$19.96** |
| 15 | Simuyemba et al., 2023 (34) | Zambia | Per dose (overall) | Financial cost | $6.00 | 2021 | *Assumed* | 1.188 | **$7.13** |
| 16 | Simuyemba et al., 2023 (34) | Zambia | Per dose (overall) | Economic cost | $23.00 | 2021 | *Assumed* | 1.188 | **$27.32** |
| 17 | Simuyemba et al., 2023 (34) | Zambia | Per FIG | Financial cost | $11.90 | 2021 | *Assumed* | 1.188 | **$14.14** |
| 18 | Simuyemba et al., 2023 (34) | Zambia | Per FIG | Economic cost | $46.00 | 2021 | *Assumed* | 1.188 | **$54.64** |
| 19 | Campos et al., 2012 (35) | Kenya/Mozambique/Tanzania/Uganda/Zimbabwe | Per dose, modelled price (low) | Vaccine price assumption | $2.00 | 2010 | *Assumed* | 1.476 | **$2.95** |
| 20 | Campos et al., 2012 (35) | Kenya/Mozambique/Tanzania/Uganda/Zimbabwe | Per dose, modelled price (high) | Vaccine price assumption | $54.25 | 2010 | *Assumed* | 1.476 | **$80.07** |
| 21 | Goldie et al., 2008 (36) | 72 GAVI-eligible countries | Per dose, modelled price | Vaccine price assumption | $2.00 | 2006 | *Assumed* | 1.597 | **$3.19** |
| 22 | Khiari et al., 2024 (37) | Tunisia | Per dose, modelled price (up to) | Vaccine price assumption | $45.00 | 2022 | *Assumed* | 1.100 | **$49.49** |
| 23 | Kiatpongsan & Kim, 2014 (38) | Kenya | Per vaccinated girl (added cost) | Vaccine price assumption | $9.70 | 2012 | *Assumed* | 1.402 | **$13.60** |
| 24 | Kiatpongsan & Kim, 2014 (38) | Uganda | Per vaccinated girl (added cost) | Vaccine price assumption | $8.30 | 2012 | *Assumed* | 1.402 | **$11.64** |
| 25 | Messoudi et al., 2019 (40) | Morocco | Per dose (vaccine price) | Vaccine price assumption | $10.00 | 2017 | *Assumed* | 1.313 | **$13.13** |
| 26 | Messoudi et al., 2019 40) | Morocco | Per dose (admin cost) | Delivery/admin cost | $5.00 | 2017 | *Assumed* | 1.313 | **$6.57** |
| 27 | Messoudi et al., 2019 (40) | Morocco | Per vaccinated girl (2-dose total) | Total assumed cost | $15.00 | 2017 | *Assumed* | 1.313 | **$19.70** |
| 28 | Slavkovsky et al., 2024 (17) | Ethiopia/Guyana/Rwanda/Sri Lanka/Uganda | Per single dose (low) | Financial cost | $4.84 | 2022 | *Assumed* | 1.100 | **$5.32** |
| 29 | Slavkovsky et al., 2024 (17) | Ethiopia/Guyana/Rwanda/Sri Lanka/Uganda | Per single dose (high) | Financial cost | $12.34 | 2022 | *Assumed* | 1.100 | **$13.57** |
| 30 | Slavkovsky et al., 2024 (17) | Ethiopia/Guyana/Rwanda/Sri Lanka/Uganda | Per 2-dose schedule (low) | Financial cost | $9.64 | 2022 | *Assumed* | 1.100 | **$10.60** |
| 31 | Slavkovsky et al., 2024 (17) | Ethiopia/Guyana/Rwanda/Sri Lanka/Uganda | Per 2-dose schedule (high) | Financial cost | $23.43 | 2022 | *Assumed* | 1.100 | **$25.77** |
| 32 | Slavkovsky et al., 2024 (17) | Ethiopia/Guyana/Rwanda/Sri Lanka/Uganda | Per single dose (low) | Economic cost | $7.86 | 2022 | *Assumed* | 1.100 | **$8.64** |
| 33 | Slavkovsky et al., 2024 (17) | Ethiopia/Guyana/Rwanda/Sri Lanka/Uganda | Per single dose (high) | Economic cost | $28.53 | 2022 | *Assumed* | 1.100 | **$31.38** |
| 34 | Burger et al., 2018 (41) | Uganda | Per dose | Assumed vaccine cost | $6.90 | 2016 | *Assumed* | 1.341 | **$9.25** |
| 35 | Burger et al., 2018 (41) | Uganda | Per 2-dose schedule | Assumed vaccine cost | $13.80 | 2016 | *Assumed* | 1.341 | **$18.51** |
| 36 | Mvundura et al., 2023 (42) | Ethiopia/Guyana/Rwanda/Senegal/Sri Lanka/Uganda | Per dose (low) | Financial cost | $0.27 | 2021 | *Assumed* | 1.188 | **$0.32** |
| 37 | Mvundura et al., 2023 (42) | Ethiopia/Guyana/Rwanda/Senegal/Sri Lanka/Uganda | Per dose (high) | Financial cost | $3.32 | 2021 | *Assumed* | 1.188 | **$3.94** |
| 38 | Mvundura et al., 2023 (42) | Ethiopia/Guyana/Rwanda/Senegal/Sri Lanka/Uganda | Per dose, Rwanda (low) | Economic cost | $3.09 | 2021 | *Assumed* | 1.188 | **$3.67** |
| 39 | Mvundura et al., 2023 (42) | Ethiopia/Guyana/Rwanda/Senegal/Sri Lanka/Uganda | Per dose (high) | Economic cost | $17.20 | 2021 | *Assumed* | 1.188 | **$20.43** |
| 40 | Quentin et al., 2012 (43) | Tanzania | Per dose, assumed vaccine price | Vaccine price assumption | $5.00 | 2010 | *Assumed* | 1.476 | **$7.38** |
| 41 | Vodicka et al., 2022 (44) | Ghana | Per dose (low) | Cost estimate | $4.50 | 2020 | *Assumed* | 1.244 | **$5.60** |
| 42 | Vodicka et al., 2022 (44) | Ghana | Per dose (high) | Cost estimate | $4.60 | 2020 | *Assumed* | 1.244 | **$5.72** |
| 43 | Umeh, Nduka & Ekwunife, 2016 (47) | Nigeria | Per dose (Gavi price) | Vaccine price assumption | $5.84 | 2014 | *Assumed* | 1.360 | **$7.94** |
| 44 | Umeh, Nduka & Ekwunife, 2016 (47) | Nigeria | Per FIG (willingness to pay) | Willingness to pay | $11.68 | 2014 | *Assumed* | 1.360 | **$15.88** |
| 45 | Biellik et al., 2009 (48) | India/Peru/Uganda/Vietnam | Per immunized child (low) | Cost estimate | $7.00 | 2007 | *Assumed* | 1.553 | **$10.87** |
| 46 | Biellik et al., 2009 (48) | India/Peru/Uganda/Vietnam | Per immunized child (high) | Cost estimate | $21.00 | 2007 | *Assumed* | 1.553 | **$32.61** |
| 47 | Portnoy et al., 2021 (53) | Ethiopia | Per dose | Cost estimate | $10.70 | 2019 | *Assumed* | 1.259 | **$13.47** |
| 48 | Levin et al., 2022 (50) | Tanzania | Per FIG | Financial cost | $6.68 | 2020 | Stated | 1.244 | **$8.31** |
| 49 | Levin et al., 2022 (50) | Tanzania | Per FIG | Economic cost | $17.31 | 2020 | Stated | 1.244 | **$21.53** |
| 50 | Hidle et al., 2022 (49) | Zimbabwe | Per dose | Financial cost | $0.53 | 2020 | *Assumed* | 1.244 | **$0.66** |
| 51 | Hidle et al., 2022 (49) | Zimbabwe | Per dose | Economic cost | $1.31 | 2020 | *Assumed* | 1.244 | **$1.63** |
| 52 | Hsiao et al., 2023 (54) | Tanzania | Per 2-dose FIG | Financial cost | $5.17 | 2021 | *Assumed* | 1.188 | **$6.14** |
| 53 | Hsiao et al., 2023 (54) | Tanzania | Per dose (1-dose scenario) | Financial cost | $2.51 | 2021 | *Assumed* | 1.188 | **$2.98** |
| 54 | Hsiao et al., 2023 (54) | Tanzania | Per 2-dose FIG | Economic cost | $22.34 | 2021 | *Assumed* | 1.188 | **$26.54** |
| 55 | Hsiao et al., 2023 (54) | Tanzania | Per dose (1-dose scenario) | Economic cost | $12.18 | 2021 | *Assumed* | 1.188 | **$14.47** |
| 56 | Mwenda et al., 2023 (52) | Kenya | Per dose (Cecolin) | Vaccine price assumption | $3.00 | 2021 | *Assumed* | 1.188 | **$3.56** |
| 57 | Mwenda et al., 2023 (52) | Kenya | Per dose (Cervarix) | Vaccine price assumption | $5.18 | 2021 | *Assumed* | 1.188 | **$6.15** |
| 58 | Mwenda et al., 2023 (52) | Kenya | Per dose (Gardasil-4) | Vaccine price assumption | $4.50 | 2021 | *Assumed* | 1.188 | **$5.35** |
| 59 | Mwenda et al., 2023 (52) | Kenya | Per dose (Gardasil-9) | Vaccine price assumption | $25.00 | 2021 | *Assumed* | 1.188 | **$29.70** |
| 60 | Mvundura et al., 2024 (51) | Ethiopia | Per dose | Financial cost | $2.23 | 2022 | *Assumed* | 1.100 | **$2.45** |
| 61 | Mvundura et al., 2024 (51) | Ethiopia | Per dose | Economic cost | $7.19 | 2022 | *Assumed* | 1.100 | **$7.91** |

FC, financial cost; EC, economic cost; FIG, fully immunized girl.

a Reference/cost year: the cost or data-collection year of the estimate. “Stated” indicates the year was explicitly reported in the source study; “Assumed” indicates the source study did not report a specific cost year and the year was estimated as publication year minus two, reflecting a conventional data-to-publication lag for costing studies. Assumed years were used as a documented placeholder and should be interpreted with this limitation in mind; where feasible, they were cross-checked against the original source.

b Basis: see footnote a.

c CPI-U deflator: ratio of the 2025 calendar-year-average CPI-U to the calendar-year-average CPI-U of the reference year, i.e. deflator = CPI₂₀₂₅ / CPI(reference year). Source: U.S. Bureau of Labor Statistics, CPI-U, U.S. city average, all items, not seasonally adjusted, calendar-year averages (cited in main text reference 51).

d Inflation-adjusted cost (2025 US$) = reported cost × CPI-U deflator. This adjustment corrects only for the erosion of US dollar purchasing power over time; it does not adjust for cross-country differences in local inflation, exchange-rate movements, or purchasing-power parity, since the original cost estimates were reported directly in US$ without the underlying local-currency and exchange-rate parameters required for such adjustment (see Discussion/Limitations).

**Methodological references (Ref number in the main text)**

25. U.S. Bureau of Labor Statistics. Consumer Price Index for All Urban Consumers (CPI-U): U.S. city average, all items [Internet]. Washington, DC: U.S. Department of Labor; 2025. (Series CUUR0000SA0). Available from: https://data.bls.gov/timeseries/CUUR0000SA0

26. Shemilt I, Thomas J, Morciano M. A web-based tool for adjusting costs to a specific target currency and price year. Evid Policy. 2010;6(1):51–59. doi:10.1332/174426410X482999

27. Husereau D, Drummond M, Augustovski F, et al. Consolidated Health Economic Evaluation Reporting Standards 2022 (CHEERS 2022). Value Health. 2022;25(1):3–9. doi:10.1016/j.jval.2021.11.1351

28. Drummond MF, Sculpher MJ, Claxton K, Stoddart GL, Torrance GW. Methods for the Economic Evaluation of Health Care Programmes. 4th ed. Oxford: Oxford University Press; 2015.
