# Supplementary material for: Financing of Human Papillomavirus Vaccination in Africa: A Scoping Review
Source: Health Sci Rep. 2026 Sep 25;9(10):e73254. doi: 10.1002/hsr2.73254 (PMC13614079; doi:10.1002/hsr2.73254)
Supplement: Supplementary file 2 [file HSR2-9-e73254-s002.docx]

**SUPPLEMENTARY FILE**

**Table S1. Search string for PubMed database search**

| **Tag** | **Subject search** | **Search String** |
| --- | --- | --- |
| #1 | Financing | ((((((((((((money[Title/Abstract]) OR (grant*[Title/Abstract])) OR (fund*[Title/Abstract])) OR (sponsor*[Title/Abstract])) OR (financ*[Title/Abstract])) OR (loan[Title/Abstract])) OR (bank*[Title/Abstract])) OR (budget*[Title/Abstract])) OR (aid[Title/Abstract])) OR (donor[Title/Abstract])) OR (donat*[Title/Abstract])) OR (invest*[Title/Abstract])) OR (allocat*[Title/Abstract]) |
| #2 | Human papillomavirus | (Human papilloma*) OR (HPV) |
| #3 | Vaccination | (vaccin*[Title/Abstract]) OR (immun*[Title/Abstract]) |
| #4 | African countries, dependencies, and territories | (((((((((((((((((((((((((((((((((((((((((((((((((((((((((((Algeria[Title/Abstract]) OR (Angola[Title/Abstract])) OR (Benin[Title/Abstract])) OR (Botswana[Title/Abstract])) OR (burkina faso[Title/Abstract])) OR (burundi[Title/Abstract])) OR (cabo verde[Title/Abstract])) OR (cape verde[Title/Abstract])) OR (cameroon[Title/Abstract])) OR (central african republic[Title/Abstract])) OR (chad[Title/Abstract])) OR (comoros[Title/Abstract])) OR (congo[Title/Abstract])) OR (ivory coast[Title/Abstract])) OR (cote d ivoire[Title/Abstract])) OR (djibouti[Title/Abstract])) OR (democratic republic of congo[Title/Abstract])) OR (egypt[Title/Abstract])) OR (equatorial guinea[Title/Abstract])) OR (eritrea[Title/Abstract])) OR (eswatini[Title/Abstract])) OR (ethiopia[Title/Abstract])) OR (gabon[Title/Abstract])) OR (gambia[Title/Abstract])) OR (ghana[Title/Abstract])) OR (guinea[Title/Abstract])) OR (guinea bissau[Title/Abstract])) OR (kenya[Title/Abstract])) OR (lesotho[Title/Abstract])) OR (liberia[Title/Abstract])) OR (libya[Title/Abstract])) OR (madagascar[Title/Abstract])) OR (malawi[Title/Abstract])) OR (mali[Title/Abstract])) OR (mauritania[Title/Abstract])) OR (mauritius[Title/Abstract])) OR (morocco[Title/Abstract])) OR (mozambique[Title/Abstract])) OR (namibia[Title/Abstract])) OR (niger[Title/Abstract])) OR (nigeria[Title/Abstract])) OR (rwanda[Title/Abstract])) OR (sao tome and principe[Title/Abstract])) OR (senegal[Title/Abstract])) OR (seychelles[Title/Abstract])) OR (sierra leone[Title/Abstract])) OR (somalia[Title/Abstract])) OR (south africa[Title/Abstract])) OR (south sudan[Title/Abstract])) OR (sudan[Title/Abstract])) OR (tanzania[Title/Abstract])) OR (togo[Title/Abstract])) OR (tunisia[Title/Abstract])) OR (uganda[Title/Abstract])) OR (zambia[Title/Abstract])) OR (zimbabwe[Title/Abstract])) OR (reunion[Title/Abstract])) OR (saint helena[Title/Abstract])) OR (western sahara[Title/Abstract])) OR (mayotte[Title/Abstract]) |
| #5 | #1 AND #2 AND #3 AND #4 | (((#1) AND (#2)) AND (#3)) AND (#4) |

**Table S2. Search string for SCOPUS database search**

| **Tag** | **Subject search** | **Search String** |
| --- | --- | --- |
| #1 | Financing | ( TITLE-ABS-KEY ( money ) OR TITLE-ABS-KEY ( grant* ) OR TITLE-ABS-KEY ( fund* ) OR TITLE-ABS-KEY ( sponsor* ) OR TITLE-ABS-KEY ( financ* ) OR TITLE-ABS-KEY ( loan ) OR TITLE-ABS-KEY ( bank* ) OR TITLE-ABS-KEY ( budget* ) OR TITLE-ABS-KEY ( aid ) OR TITLE-ABS-KEY ( donor ) OR TITLE-ABS-KEY ( donat* ) OR TITLE-ABS-KEY ( invest* ) OR TITLE-ABS-KEY ( allocat* ) ) |
| #2 | Human papillomavirus | ( ALL ( "human papilloma*" ) OR ALL ( hpv ) ) |
| #3 | Vaccination | ( TITLE-ABS-KEY ( vaccin* ) OR TITLE-ABS-KEY ( immun* ) ) |
| #4 | African countries, dependencies, and territories | ( ( TITLE-ABS-KEY ( angola )  OR  TITLE-ABS-KEY ( benin )  OR  TITLE-ABS-KEY ( botswana )  OR  TITLE-ABS-KEY ( "burkina faso" )  OR  TITLE-ABS-KEY ( burundi )  OR  TITLE-ABS-KEY ( cameroon )  OR  TITLE-ABS-KEY ( "cabo verde" )  OR  TITLE-ABS-KEY ( "cape verde" )  OR  TITLE-ABS-KEY ( "central african republic" )  OR  TITLE-ABS-KEY ( chad )  OR  TITLE-ABS-KEY ( comoros )  OR  TITLE-ABS-KEY ( congo )  OR  TITLE-ABS-KEY ( "ivory coast" )  OR  TITLE-ABS-KEY ( "democratic republic of congo" )  OR  TITLE-ABS-KEY ( djibouti )  OR  TITLE-ABS-KEY ( "equatorial guinea" )  OR  TITLE-ABS-KEY ( eritrea )  OR  TITLE-ABS-KEY ( ethiopia )  OR  TITLE-ABS-KEY ( gabon )  OR  TITLE-ABS-KEY ( gambia )  OR  TITLE-ABS-KEY ( ghana )  OR  TITLE-ABS-KEY ( guinea )  OR  TITLE-ABS-KEY ( guinea-bissau )  OR  TITLE-ABS-KEY ( kenya )  OR  TITLE-ABS-KEY ( lesotho )  OR  TITLE-ABS-KEY ( liberia )  OR  TITLE-ABS-KEY ( madagascar )  OR  TITLE-ABS-KEY ( malawi )  OR  TITLE-ABS-KEY ( mali )  OR  TITLE-ABS-KEY ( mauritania )  OR  TITLE-ABS-KEY ( mauritius )  OR  TITLE-ABS-KEY ( mayotte )  OR  TITLE-ABS-KEY ( mozambique )  OR  TITLE-ABS-KEY ( namibia )  OR  TITLE-ABS-KEY ( niger )  OR  TITLE-ABS-KEY ( nigeria )  OR  TITLE-ABS-KEY ( reunion )  OR  TITLE-ABS-KEY ( rwanda )  OR  TITLE-ABS-KEY ( "saint helena" )  OR  TITLE-ABS-KEY ( sao  AND tome  AND  principe )  OR  TITLE-ABS-KEY ( senegal )  OR  TITLE-ABS-KEY ( seychelles )  OR  TITLE-ABS-KEY ( "sierra leone" )  OR  TITLE-ABS-KEY ( somalia )  OR  TITLE-ABS-KEY ( "south africa" )  OR  TITLE-ABS-KEY ( "south sudan" ) ) )  OR  ( ( TITLE-ABS-KEY ( eswatini )  OR  TITLE-ABS-KEY ( togo )  OR  TITLE-ABS-KEY ( uganda )  OR  TITLE-ABS-KEY ( zambia )  OR  TITLE-ABS-KEY ( zimbabwe )  OR  TITLE-ABS-KEY ( egypt )  OR  TITLE-ABS-KEY ( libya )  OR  TITLE-ABS-KEY ( algeria )  OR  TITLE-ABS-KEY ( tunisia )  OR  TITLE-ABS-KEY ( morocco )  OR  TITLE-ABS-KEY ( "western sahara" )  OR  TITLE-ABS-KEY ( sudan )  OR  TITLE-ABS-KEY ( tunisia ) ) ) |
| #5 | #1 AND #2 AND #3 AND #4 | ( ( ALL ( "human papilloma*" ) OR ALL ( hpv ) ) ) AND ( ( ( ( TITLE-ABS-KEY ( angola ) OR TITLE-ABS-KEY ( benin ) OR TITLE-ABS-KEY ( botswana ) OR TITLE-ABS-KEY ( "burkina faso" ) OR TITLE-ABS-KEY ( burundi ) OR TITLE-ABS-KEY ( cameroon ) OR TITLE-ABS-KEY ( "cabo verde" ) OR TITLE-ABS-KEY ( "cape verde" ) OR TITLE-ABS-KEY ( "central african republic" ) OR TITLE-ABS-KEY ( chad ) OR TITLE-ABS-KEY ( comoros ) OR TITLE-ABS-KEY ( congo ) OR TITLE-ABS-KEY ( "ivory coast" ) OR TITLE-ABS-KEY ( "democratic republic of congo" ) OR TITLE-ABS-KEY ( djibouti ) OR TITLE-ABS-KEY ( "equatorial guinea" ) OR TITLE-ABS-KEY ( eritrea ) OR TITLE-ABS-KEY ( ethiopia ) OR TITLE-ABS-KEY ( gabon ) OR TITLE-ABS-KEY ( gambia ) OR TITLE-ABS-KEY ( ghana ) OR TITLE-ABS-KEY ( guinea ) OR TITLE-ABS-KEY ( guinea-bissau ) OR TITLE-ABS-KEY ( kenya ) OR TITLE-ABS-KEY ( lesotho ) OR TITLE-ABS-KEY ( liberia ) OR TITLE-ABS-KEY ( madagascar ) OR TITLE-ABS-KEY ( malawi ) OR TITLE-ABS-KEY ( mali ) OR TITLE-ABS-KEY ( mauritania ) OR TITLE-ABS-KEY ( mauritius ) OR TITLE-ABS-KEY ( mayotte ) OR TITLE-ABS-KEY ( mozambique ) OR TITLE-ABS-KEY ( namibia ) OR TITLE-ABS-KEY ( niger ) OR TITLE-ABS-KEY ( nigeria ) OR TITLE-ABS-KEY ( reunion ) OR TITLE-ABS-KEY ( rwanda ) OR TITLE-ABS-KEY ( "saint helena" ) OR TITLE-ABS-KEY ( sao AND tome AND principe ) OR TITLE-ABS-KEY ( senegal ) OR TITLE-ABS-KEY ( seychelles ) OR TITLE-ABS-KEY ( "sierra leone" ) OR TITLE-ABS-KEY ( somalia ) OR TITLE-ABS-KEY ( "south africa" ) OR TITLE-ABS-KEY ( "south sudan" ) ) ) OR ( ( TITLE-ABS-KEY ( eswatini ) OR TITLE-ABS-KEY ( togo ) OR TITLE-ABS-KEY ( uganda ) OR TITLE-ABS-KEY ( zambia ) OR TITLE-ABS-KEY ( zimbabwe ) OR TITLE-ABS-KEY ( egypt ) OR TITLE-ABS-KEY ( libya ) OR TITLE-ABS-KEY ( algeria ) OR TITLE-ABS-KEY ( tunisia ) OR TITLE-ABS-KEY ( morocco ) OR TITLE-ABS-KEY ( "western sahara" ) OR TITLE-ABS-KEY ( sudan ) OR TITLE-ABS-KEY ( tunisia ) ) ) ) AND ( ( TITLE-ABS-KEY ( "human papilloma*" ) OR TITLE-ABS-KEY ( hpv ) ) ) AND ( ( TITLE-ABS-KEY ( superstit* ) OR TITLE-ABS-KEY ( fallacy ) OR TITLE-ABS-KEY ( delusion ) OR TITLE-ABS-KEY ( misconception ) OR TITLE-ABS-KEY ( fantasy ) OR TITLE-ABS-KEY ( falsehood ) OR TITLE-ABS-KEY ( falsity ) OR TITLE-ABS-KEY ( "false notion" ) OR TITLE-ABS-KEY ( "irrational belief" ) ) ) ) AND ( ( TITLE-ABS-KEY ( vaccin* ) OR TITLE-ABS-KEY ( immun* ) ) ) AND ( ( TITLE-ABS-KEY ( money ) OR TITLE-ABS-KEY ( grant* ) OR TITLE-ABS-KEY ( fund* ) OR TITLE-ABS-KEY ( sponsor* ) OR TITLE-ABS-KEY ( financ* ) OR TITLE-ABS-KEY ( loan ) OR TITLE-ABS-KEY ( bank* ) OR TITLE-ABS-KEY ( budget* ) OR TITLE-ABS-KEY ( aid ) OR TITLE-ABS-KEY ( donor ) OR TITLE-ABS-KEY ( donat* ) OR TITLE-ABS-KEY ( invest* ) OR TITLE-ABS-KEY ( allocat* ) ) ) |

**Table S3. Search string for other database (AMED – The Allied and Complementary Medicine Database, CINAHL Ultimate, Dentistry and Oral Sciences Source, SPORTDiscus with Full Text, APA PsycArticles, Psychology and Behavioral Sciences Collection, and APA PsycInfo) search via EBSCO interface**

| **Tag** | **Subject search** | **Search String** |
| --- | --- | --- |
| S1 | Financing | AB money OR AB grant* OR AB fund* OR AB sponsor* OR AB financ* OR AB loan OR AB bank* OR AB budget* OR AB aid OR AB donor OR AB donat* OR AB invest* OR AB allocat* |
| S2 | Human papillomavirus | Human papilloma* OR HPV |
| S3 | Vaccination | AB vaccin* OR AB immune* |
| S4 | African countries, dependencies, and territories | AB algeria OR AB angola OR AB benin OR AB botswana OR AB burkina faso OR AB burundi OR AB cape verde OR AB cabo verde OR AB cameroon OR AB central african republic OR AB chad OR AB comoros OR AB congo OR AB cote d'ivoire OR AB ivory coast OR AB djibouti OR AB democratic republic of congo OR AB egypt OR AB equatorial guinea OR AB eritrea OR AB eswatini OR AB ethiopia OR AB gabon OR AB gambia OR AB ghana OR AB guinea OR AB guinea bissau OR AB kenya OR AB lesotho OR AB liberia OR AB libya OR AB madagascar OR AB malawi OR AB mali OR AB mauritania OR AB mauritius OR AB morocco OR AB mozambique OR AB namibia OR AB niger OR AB nigeria OR AB rwanda OR AB ( sao tome and principe ) OR AB senegal OR AB seychelles OR AB sierra leone OR AB somalia OR AB south Africa OR AB south sudan OR AB sudan OR AB tanzania OR AB togo OR AB tunisia OR AB uganda OR AB zambia OR AB zimbabwe OR AB reunion OR AB saint helena OR AB western sahara OR AB mayotte |
| S5 | S1 AND S2 AND S3 AND S4 | S1 AND S2 AND S3 AND S4 |

**TABLE S4. List of articles whose full texts were screened for inclusion/exclusion into the scoping review.**

| **No.** | **CITATION** | **INCLUDED** | **EXCLUDED (REASONS)** |
| --- | --- | --- | --- |
| 1 | Alonso, S., Cambaco, O., Maússe, Y., Matsinhe, G., Macete, E., Menéndez, C., Sicuri, E., Sevene, E., & Munguambe, K. (2019). Costs associated with delivering HPV vaccination in the context of the first year demonstration programme in southern Mozambique. *BMC Public Health*, *19*(1), 1031. https://doi.org/10.1186/s12889-019-7338-4 | Yes |  |
| 2 | Bencina, G., Ugrekhelidze, D., Shoel, H., Oliver, E., Meiwald, A., Hughes, R., Eiden, A., & Weston, G. (2024). The indirect costs of vaccine-preventable cancer mortality in the Middle East and North Africa (MENA). *Journal of Medical Economics*, *27*(sup2), 41–50. https://doi.org/10.1080/13696998.2024.2384264 | No | Wrong design |
| 3 | Biellik, R., Levin, C., Mugisha, E., LaMontagne, D. S., Bingham, A., Kaipilyawar, S., & Gandhi, S. (2009). Health systems and immunization financing for human papillomavirus vaccine introduction in low-resource settings. *Vaccine*, *27*(44), 6203–6209. https://doi.org/10.1016/j.vaccine.2009.08.003 | Yes |  |
| 4 | Brennan, T., Hidle, A., Doshi, R. H., An, Q., Loharikar, A., Casey, R., Badiane, O., Ndiaye, A., Diallo, A., Loko Roka, J., Mejia, N., & Abimbola, T. (2022). Cost of human papillomavirus vaccine delivery in a single-age cohort, routine-based vaccination program in Senegal. *Vaccine*, *40*, A77–A84. https://doi.org/10.1016/j.vaccine.2021.11.057 | Yes |  |
| 5 | Burger, E. A., Campos, N. G., Sy, S., Regan, C., & Kim, J. J. (2018). Health and economic benefits of single-dose HPV vaccination in a Gavi-eligible country. *Vaccine*, *36*(32), 4823–4829. https://doi.org/10.1016/j.vaccine.2018.04.061 | Yes |  |
| 6 | Campos, N. G., Kim, J. J., Castle, P. E., Ortendahl, J. D., O’Shea, M., Diaz, M., & Goldie, S. J. (2012). Health and economic impact of HPV 16/18 vaccination and cervical cancer screening in Eastern Africa. *International Journal of Cancer*, *130*(11), 2672–2684. https://doi.org/10.1002/ijc.26269 | Yes |  |
| 7 | Chigozie, N., Hilfinger Messiaa, D. K., Adebola, A., & Ojiegbe, T. (2022). Men’s willingness to support HPV vaccination and cervical cancer screening in Nigeria. *Health Promotion International*, *37*(1), daab056. https://doi.org/10.1093/heapro/daab056 | Yes |  |
| 8 | Goldie, S. J., O’Shea, M., Campos, N. G., Diaz, M., Sweet, S., & Kim, S.-Y. (2008). Health and economic outcomes of HPV 16,18 vaccination in 72 GAVI-eligible countries. *Vaccine*, *26*(32), 4080–4093. https://doi.org/10.1016/j.vaccine.2008.04.053 | Yes |  |
| 9 | Hidle, A., Brennan, T., Garon, J., An, Q., Loharikar, A., Marembo, J., Manangazira, P., Mejia, N., & Abimbola, T. (2022). Cost of human papillomavirus vaccine delivery at district and health facility levels in Zimbabwe: A school-based vaccination program targeting multiple cohorts. *Vaccine*, *40*, A67–A76. https://doi.org/10.1016/j.vaccine.2022.01.024 | Yes |  |
| 10 | Hidle, A., Gwati, G., Abimbola, T., Pallas, S. W., Hyde, T., Petu, A., McFarland, D., & Manangazira, P. (2018). Cost of a human papillomavirus vaccination project, Zimbabwe. *Bulletin of the World Health Organization*, *96*(12), 834–842. https://doi.org/10.2471/BLT.18.211904 | Yes |  |
| 11 | Hsiao, A., Struckmann, V., Stephani, V., Mmbando, D., Changalucha, J., Baisley, K., Levin, A., Morgan, W., Hutubessy, R., Watson – Jones, D., Whitworth, H., & Quentin, W. (2023). Costs of delivering human papillomavirus vaccination using a one- or two-dose strategy in Tanzania. *Vaccine*, *41*(2), 372–379. https://doi.org/10.1016/j.vaccine.2022.11.032 | Yes |  |
| 12 | Hutubessy, R., Levin, A., Wang, S., Morgan, W., Ally, M., John, T., & Broutet, N. (2012). A case study using the United Republic of Tanzania: Costing nationwide HPV vaccine delivery using the WHO Cervical Cancer Prevention and Control Costing Tool. *BMC Medicine*, *10*(1), 136. https://doi.org/10.1186/1741-7015-10-136 | Yes |  |
| 13 | Khiari, H., Makni, K., Meddeb, K., Jaidane, O., & Hsairi, M. (2024). Cost-effectiveness of human papillomavirus (HPV) vaccination in Tunisia: A modelling study. *BMJ Open*, *14*(12), e085462. https://doi.org/10.1136/bmjopen-2024-085462 | Yes |  |
| 14 | Kiatpongsan, S., & Kim, J. J. (2014). Costs and Cost-Effectiveness of 9-Valent Human Papillomavirus (HPV) Vaccination in Two East African Countries. *PLoS ONE*, *9*(9), e106836. https://doi.org/10.1371/journal.pone.0106836 | Yes |  |
| 15 | Kiendrébéogo, J. A., Sidibe, A. R. O., Compaoré, G. B., Nacanabo, R., Sory, O., Ouédraogo, I., Nawaz, S., Schuind, A. E., & Clark, A. (2023). Cost-effectiveness of human papillomavirus (HPV) vaccination in Burkina Faso: A modelling study. *BMC Health Services Research*, *23*(1), 1338. https://doi.org/10.1186/s12913-023-10283-3 | Yes |  |
| 16 | Levin, A., Yuma, S., Swai, E., Morgan, W., Gauvreau, C. L., Broutet, N., Yeung, K. H. T., & Hutubessy, R. (2022). Comprehensive approach to costing cervical cancer prevention and control: A case study in the United Republic of Tanzania using the Cervical Cancer Prevention and Control Costing (C4P) tool. *BMC Medicine*, *20*(1), 384. https://doi.org/10.1186/s12916-022-02576-x | Yes |  |
| 17 | Marfo, E., Salami, B., Adjei, C., & MacDonald, S. (2024). Human papillomavirus (HPV) vaccination in a privately funded program in Ghana: A qualitative case study. *Human Vaccines & Immunotherapeutics*, *20*(1), 2397219. https://doi.org/10.1080/21645515.2024.2397219 | Yes |  |
| 18 | Messoudi, W., Elmahi, T., Nejjari, C., Tachfouti, N., Zidouh, A., Saadani, G., Moriña, D., & Diaz, M. (2019). Cervical cancer prevention in Morocco: A model-based cost-effectiveness analysis. *Journal of Medical Economics*, *22*(11), 1153–1159. https://doi.org/10.1080/13696998.2019.1624556 | Yes |  |
| 19 | Mvundura, M., Bayeh, A., Zelalem, M., Lakew, Y., Dhufera, A., Dagnew, B., Slavkovsky, R., & Lamontagne, D. S. (2024). Evaluating the cost and operational context for national human papillomavirus (HPV) vaccine delivery in three regions of Ethiopia. *PLOS Global Public Health*, *4*(7), e0003357. https://doi.org/10.1371/journal.pgph.0003357 | Yes |  |
| 20 | Mvundura, M., Slavkovsky, R., Debellut, F., Naddumba, T., Bayeh, A., Ndiaye, C., Anena, J., Vodicka, E., Diop, A., Gamage, D., Musanabaganwa, C., Tatkan, G., Driwale, A., Zelalem, M., Badiane, O., Ginige, S., Hamilton, E., Sibomana, H., Lakew, Y., … Lamontagne, D. S. (2023). Cost and operational context for national human papillomavirus (HPV) vaccine delivery in six low- and middle-income countries. *Vaccine*, *41*(49), 7435–7443. https://doi.org/10.1016/j.vaccine.2023.11.008 | Yes |  |
| 21 | Mwenda, V., Jalang’o, R., Miano, C., Bor, J.-P., Nyangasi, M., Mecca, L., Were, V., Kariithi, E., Pecenka, C., Schuind, A., Abbas, K., & Clark, A. (2023). Impact, cost-effectiveness, and budget implications of HPV vaccination in Kenya: A modelling study. *Vaccine*, *41*(29), 4228–4238. https://doi.org/10.1016/j.vaccine.2023.05.019 | Yes |  |
| 22 | Ngabo, F., Levin, A., Wang, S. A., Gatera, M., Rugambwa, C., Kayonga, C., Donnen, P., Lepage, P., & Hutubessy, R. (2015). A cost comparison of introducing and delivering pneumococcal, rotavirus and human papillomavirus vaccines in Rwanda. *Vaccine*, *33*(51), 7357–7363. https://doi.org/10.1016/j.vaccine.2015.10.022 | Yes |  |
| 23 | Portnoy, A., Sweet, S., Desalegn, D., Memirie, S. T., Kim, J. J., & Verguet, S. (2021). Health gains and financial protection from human papillomavirus vaccination in Ethiopia: Findings from a modelling study. *Health Policy and Planning*, *36*(6), 891–899. https://doi.org/10.1093/heapol/czab052 | Yes |  |
| 24 | Quentin, W., Terris-Prestholt, F., Changalucha, J., Soteli, S., Edmunds, W. J., Hutubessy, R., Ross, D. A., Kapiga, S., Hayes, R., & Watson-Jones, D. (2012). Costs of delivering human papillomavirus vaccination to schoolgirls in Mwanza Region, Tanzania. *BMC Medicine*, *10*(1), 137. https://doi.org/10.1186/1741-7015-10-137 | Yes |  |
| 25 | Slavkovsky, R., Mvundura, M., Debellut, F., & Naddumba, T. (2024). Evaluating potential program cost savings with a single-dose HPV vaccination schedule: A modeling study. *JNCI Monographs*, *2024*(67), 371–378. https://doi.org/10.1093/jncimonographs/lgae037 | Yes |  |
| 26 | The Lancet. (2011). Financing HPV vaccination in developing countries. *The Lancet*, *377*(9777), 1544. https://doi.org/10.1016/S0140-6736(11)60622-3 | No | Wrong design |
| 27 | Umeh, I. B., Nduka, S. O., & Ekwunife, O. I. (2016). Mothers’ willingness to pay for HPV vaccines in Anambra state, Nigeria: A cross sectional contingent valuation study. *Cost Effectiveness and Resource Allocation*, *14*(1), 8. https://doi.org/10.1186/s12962-016-0057-0 | Yes |  |
| 28 | Vodicka, E., Nonvignon, J., Antwi-Agyei, K. O., Bawa, J., Clark, A., Pecenka, C., & LaMontagne, D. S. (2022). The projected cost-effectiveness and budget impact of HPV vaccine introduction in Ghana. *Vaccine*, *40*, A85–A93. https://doi.org/10.1016/j.vaccine.2021.07.027 | Yes |  |
| 29 | Simuyemba MC, Chama-Chiliba CM, Chompola A, Sinyangwe A, Bchir A, Asiimwe G, Masiye F, & Chibwesha C. (2023). An Evaluation of the Cost of Human Papilloma Virus (HPV) Vaccine Delivery In Zambia. *Research Square*. <https://doi.org/10.21203/rs.3.rs-2919637/v1> | Yes |  |

**Table S5. Quality appraisal outcomes of the appraised qualitative study using the Mixed Methods Appraisal Tool**

| **No.** | **Author (Year)** | **Study Design** | **Responses to the Appraisal Questions for Qualitative Studies** | | | | | | | **Scored Points (out of a Total of 7 Points)** | **Grade** |
| --- | --- | --- | --- | --- | --- | --- | --- | --- | --- | --- | --- |
|  |  |  | Are there clear research questions? | Do the collected data allow to address the research questions? | Is the qualitative approach appropriate to answer the research question? | Are the qualitative data collection methods adequate to address the research question? | Are the findings adequately derived from the data? | Is the interpretation of results sufficiently substantiated by data? | Is there coherence between qualitative data sources, collection, analysis and interpretation? |  |  |
| 1 | Biellik et al., (2009) | Qualitative descriptive study | Yes | Yes | Yes | Yes | Yes | Yes | Yes | 7/7 | Above Average |
| 2 | Marfo et al., (2024) | Qualitative descriptive study | Yes | Yes | Yes | Yes | Yes | Yes | Yes | 7/7 | Above Average |
| Yes – 1 point; No – 0 point; I can’t tell – 0.5 point; Average – 3.5 points; Above average – >3.5 points; Below average – <3.5 points | | | | | | | | | | | |

**Table S6. Quality appraisal outcomes of the appraised quantitative randomized studies using the Mixed Methods Appraisal Tool**

| **No.** | **Author (Year)** | **Study Design** | **Responses to the Appraisal Questions for Quantitative Randomized Studies** | | | | | | | **Scored Points (out of a Total of 7 Points)** | **Grade** |
| --- | --- | --- | --- | --- | --- | --- | --- | --- | --- | --- | --- |
|  |  |  | Are there clear research questions? | Do the collected data allow to address the research questions? | Is randomization appropriately performed? | Are the groups comparable at baseline? | Are there complete outcome data? | Are outcome assessors blinded to the intervention provided? | Did the participants adhere to the assigned intervention? |  |  |
| 1 | Nil | Nil | Nil | Nil | Nil | Nil | Nil | Nil | Nil | Nil | Nil |
| Yes – 1 point; No – 0 point; I can’t tell – 0.5 point; Average – 3.5 points; Above average – >3.5 points; Below average – <3.5 points | | | | | | | | | | | |

**Table S7. Quality appraisal outcomes of the appraised quantitative non-randomized studies using the Mixed Methods Appraisal Tool**

| **No.** | **Author (Year)** | **Study**  **Design** | **Responses to the Appraisal Questions for Quantitative Non-randomized Studies** | | | | | | | **Scored Points (out of a Total of 7 Points)** | **Grade** |
| --- | --- | --- | --- | --- | --- | --- | --- | --- | --- | --- | --- |
|  |  |  | Are there clear research questions? | Do the collected data allow to address the research questions? | Are the participants representative of the largest population? | Are measurements appropriate regarding both the outcome and intervention (or exposure)? | Are there complete outcome data? | Are the confounders accounted for in the design and analysis? | During the study period, is the intervention administered (or exposure occurred) as intended? |  |  |
| 1. | Brennan et al., (2022) | Cohort study (Retrospective) | Yes | Yes | I can’t tell | Yes | I can’t tell | Yes | Yes | 6/7 | Above Average |
| 2. | Chigozie et al., (2022) | Cross-sectional analytical study | Yes | Yes | I can’t tell | Yes | No | Yes | Yes | 5.5/7 | Above Average |
| 3. | Hidle et al., (2022) | Cohort study (Retrospective) | Yes | Yes | I can’t tell | Yes | I can’t tell | Yes | Yes | 6/7 | Above Average |
| 4. | Umeh et al., (2016) | Cross-sectional analytical study | Yes | Yes | No | Yes | Yes | I can’t tell | No | 4.5/7 | Above Average |
| Yes – 1 point; No – 0 point; I can’t tell – 0.5 point; Average – 3.5 points; Above average – >3.5 points; Below average – <3.5 points | | | | | | | | | | | |

**Table S8. Quality appraisal outcomes of the appraised quantitative descriptive study using the Mixed Methods Appraisal Tool**

| **No.** | **Author (Year)** | **Study Design** | **Responses to the Appraisal Questions for Quantitative Descriptive Studies** | | | | | | | **Scored Points (out of a Total of 7 Points)** | **Grade** |
| --- | --- | --- | --- | --- | --- | --- | --- | --- | --- | --- | --- |
|  |  |  | Are there clear research questions? | Do the collected data allow to address the research questions? | Is the sampling strategy relevant to address the research question? | Is the sample representative of the target population? | Are the measurements appropriate? | Is the risk of nonresponse bias low? | Is the statistical analysis appropriate to answer the research question? |  |  |
| 1. | Burger et al., (2018) | Quantitative Descriptive Study | Yes | Yes | Yes | Yes | Yes | No | Yes | 6/7 | Above Average |
| 2. | Campos et al., (2012) | Quantitative Descriptive Study | Yes | Yes | Yes | Yes | Yes | No | Yes | 6/7 | Above Average |
| 3. | Goldie et al., (2008) | Quantitative Descriptive Study | Yes | Yes | Yes | Yes | Yes | No | Yes | 6/7 | Above Average |
| 4. | Hidle et al., (2018) | Quantitative Descriptive Study | Yes | Yes | Yes | Yes | Yes | Yes | Yes | 6/7 | Above Average |
| 5. | Hutubessy et al., (2012) | Quantitative Descriptive Study | Yes | Yes | No | No | Yes | No | Yes | 4/7 | Above Average |
| 6. | Khiari et al., (2024) | Quantitative Descriptive Study | Yes | Yes | Yes | Yes | Yes | No | Yes | 6/7 | Above Average |
| 7. | Kiatpongsan & Kim, (2014) | Quantitative Descriptive Study | Yes | Yes | Yes | Yes | Yes | No | Yes | 6/7 | Above Average |
| 8. | Kiendrébéogo et al., (2023) | Quantitative Descriptive Study | Yes | Yes | Yes | Yes | Yes | No | Yes | 6/7 | Above Average |
| 9. | Levin et al., (2022) | Quantitative Descriptive Study | Yes | Yes | Yes | Yes | Yes | No | Yes | 6/7 | Above Average |
| 10. | Ngabo et al., (2015) | Quantitative Descriptive Study | Yes | Yes | Yes | I can’t tell | Yes | I can’t tell | Yes | 6/7 | Above Average |
| 11. | Portnoy et al., (2021) | Quantitative Descriptive Study | Yes | Yes | Yes | I can’t tell | Yes | I can’t tell | Yes | 6/7 | Above Average |
| 12. | Vodicka et al., (2022) | Quantitative Descriptive Study | Yes | Yes | I can’t tell | I can’t tell | Yes | No | Yes | 5/7 | Above Average |
| 13. | Simuyemba et al., (2023) | Quantitative Descriptive Study | Yes | Yes | Yes | Yes | Yes | Yes | Yes | 7/7 | Above Average |
| 14. | Messoudi et al., (2019) | Quantitative Descriptive Study | Yes | Yes | Yes | Yes | Yes | No | Yes | 6/7 | Above Average |
| 15. | Mwenda et al., (2023) | Quantitative Descriptive Study | Yes | Yes | Yes | Yes | Yes | No | Yes | 6/7 | Above Average |
| 16. | Quentin et al., (2012) | Quantitative Descriptive Study | Yes | Yes | Yes | Yes | Yes | No | Yes | 6/7 | Above Average |
| 17. | Slavkovsky et al., (2024) | Quantitative Descriptive Study | Yes | Yes | Yes | Yes | Yes | No | Yes | 6/7 | Above Average |
| Yes – 1 point; No – 0 point; I can’t tell – 0.5 point; Average – 3.5 points; Above average – >3.5 points; Below average – <3.5 points | | | | | | | | | | | |

**Table S9. Quality appraisal outcomes of the appraised Mixed-method study design using the Mixed Methods Appraisal Tool**

| **No.** | **Author (Year)** | **Study Design** | **Responses to the Appraisal Questions for Mixed-method study designs** | | | | | | | **Scored Points (out of a Total of 7 Points)** | **Grade** |
| --- | --- | --- | --- | --- | --- | --- | --- | --- | --- | --- | --- |
|  |  |  | Are there clear research questions? | Do the collected data allow to address the research questions? | Is there an adequate rationale for using a mixed methods design to address the research question? | Are the different components of the study effectively integrated to answer the research question? | Are the outputs of the integration of qualitative and quantitative components adequately interpreted? | Are divergences and inconsistencies between quantitative and qualitative results adequately addressed? | Do the different components of the study adhere to the quality criteria of each tradition of the methods involved? |  |  |
| 1 | Alonso et al. (2019) | Mixed-method Study | Yes | Yes | Yes | Yes | Yes | I can’t tell | Yes | 6.5/7 | Above Average |
| 2 | Hsiao et al., (2023) | Mixed Methods Study | Yes | Yes | I can’t tell | Yes | Yes | No | Yes | 5.5/7 | Above Average |
| 3 | Mvundura et al., (2024) | Mixed Methods Study | Yes | Yes | Yes | Yes | Yes | Yes | Yes | 7/7 | Above Average |
| 4 | Mvundura et al., (2023) | Mixed Methods Study | Yes | Yes | Yes | Yes | Yes | Yes | Yes | 7/7 | Above Average |
| Yes – 1 point; No – 0 point; I can’t tell – 0.5 point; Average – 3.5 points; Above average – >3.5 points; Below average – <3.5 points | | | | | | | | | | | |
